# Conversion of Metal Pyrazolate/(Hydr)oxide Clusters into Nanojars: Solution vs Solid-State Structure and Magnetism

**DOI:** 10.1021/acs.inorgchem.4c01698

**Published:** 2024-06-14

**Authors:** Pooja Singh, Wisam A. Al Isawi, Matthias Zeller, Gellert Mezei

**Affiliations:** † Department of Chemistry, Western Michigan University, Kalamazoo, Michigan 49008, United States; ‡ Department of Chemistry, Purdue University, West Lafayette, Indiana 47907, United States

## Abstract

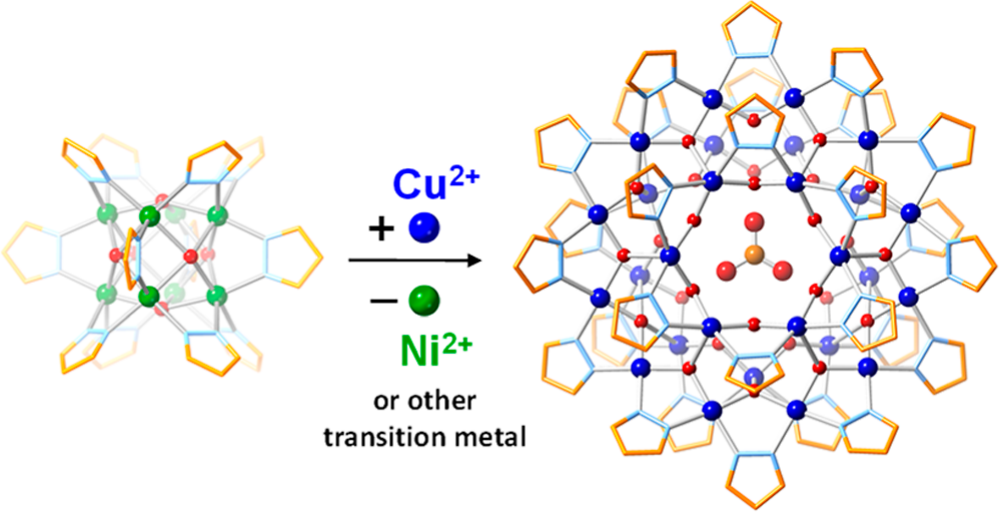

Nanojars are a class of anion binding and extraction agents composed of a series of [Cu(μ-OH)(μ-pz)]_*n*_ (pz = pyrazolate; *n* = 26–36) supramolecular metal–organic complexes. In contrast to other anion binding agents amenable to liquid–liquid extraction, nanojars only form by self-assembly around the target anion, and guest-free nanojar hosts cannot be isolated. An extraordinary binding strength toward highly hydrophilic anions such as carbonate and sulfate was demonstrated by the inability of Ba^2+^ ions to precipitate the corresponding insoluble barium salts from nanojars. Herein, we provide an additional proof for the superior robustness of the nanojar framework based on competition experiments with other transition metal pyrazolate/(hydr)oxide complexes. In addition to the mass spectrometric characterization, we present variable-temperature nuclear magnetic resonance studies with an emphasis on the influence of the paramagnetic Cu^2+^ centers on ^1^H hyperfine shifts, along with X-ray crystallographic analysis of two polymorphs of (MePh_3_P)_2_[CO_3_⊂{Cu(OH)(pz)}_27_], including the highest (cubic) symmetry nanojar crystal lattice obtained to date as well as magnetism studies for the first time. Furthermore, we provide evidence for the first molybdate-incarcerating nanojars, [MoO_4_⊂{Cu(μ-OH)(μ-pz)}_*n*_]^2–^ (*n* = 28, 31–33), formed by rearrangement from [Mo^VI^_8_O_12_(μ-O)_9_(μ-pz)_6_(pzH)_6_·3pzH] in the presence of Cu^2+^ ions.

## Abstract 2

Transition metal pyrazolate/(hydr)oxide complexes transform into more robust nanojars in the presence of Cu^2+^ ions and incarcerate highly hydrophilic, inorganic oxoanions. The structure of nanojars in the solid state (by X-ray crystallography) and in solution (by ^1^H NMR spectroscopy) are contrasted, and their magnetism is studied.

## Introduction

Nanojars are a unique class of copper complexes that self-assemble from Cu(OH)_2_ and pyrazole by wrapping around and incarcerating anions.^1^ More generally, nanojars of the formula [anion⊂{*cis*-Cu^II^(μ-OH)(μ-pz)}_*n*_]^2–^ (Cu_*n*_; *n* = 26–36; pz = pyrazolate ion, C_3_H_3_N_2_^–^) form whenever a mixture of Cu^2+^ ions and pyrazole (or a C-substituted derivative)^2^ is treated with a strong base, such as NaOH or Bu_4_NOH, which easily absorbs CO_2_ from the air and converts it to carbonate.^3−5^ The three (or four)^1^ charge-neutral constituent [*cis*-Cu^II^(μ-OH)(μ-pz)]_*x*_ (Cu_*x*_; *x* = 6–14, except 11) metallamacrocycles (rings) of nanojars are bound together by a multitude of hydrogen bonds and axial Cu–O interactions. The anion bound in the cavity of the nanojar by hydrogen bonding to its two smaller side rings appears to be an essential, additional supramolecular “glue” that ultimately allows for the formation of the nanojar host because an empty, guest-free nanojar could never be observed. As anion binding and extraction agents, nanojars exhibit an inverse Hofmeister bias^6,7^ and preferentially bind anions with large hydration energies, such as CO_3_^2–^ (Δ*G*_h_° = −1324 kJ/mol) and SO_4_^2–^ (Δ*G*_h_° = −1064 kJ/mol).^8^ As measurements of binding constants by host–guest titration are precluded by the inability to isolate the empty host, the strength of anion binding by nanojars was assessed by competitive binding studies using Ba^2+^. Experiments conducted under both heterogeneous [using an aqueous solution of Ba(NO_3_)_2_ and an immiscible, dichloromethane or 2-methyltetrahydrofuran solution of nanojars] and homogeneous (using 2-methyltetrahydrofuran solutions of barium dioctyl sulfosuccinate and nanojars) conditions suggest very strong binding^3,^^9^ as the incarcerated anion did not precipitate out as the highly insoluble BaCO_3_ or BaSO_4_ salts (*K*_sp_ = 2.58 × 10^–9^ and 1.08 × 10^–10^, respectively, in H_2_O at 25 °C).^10^

**Figure 1:**
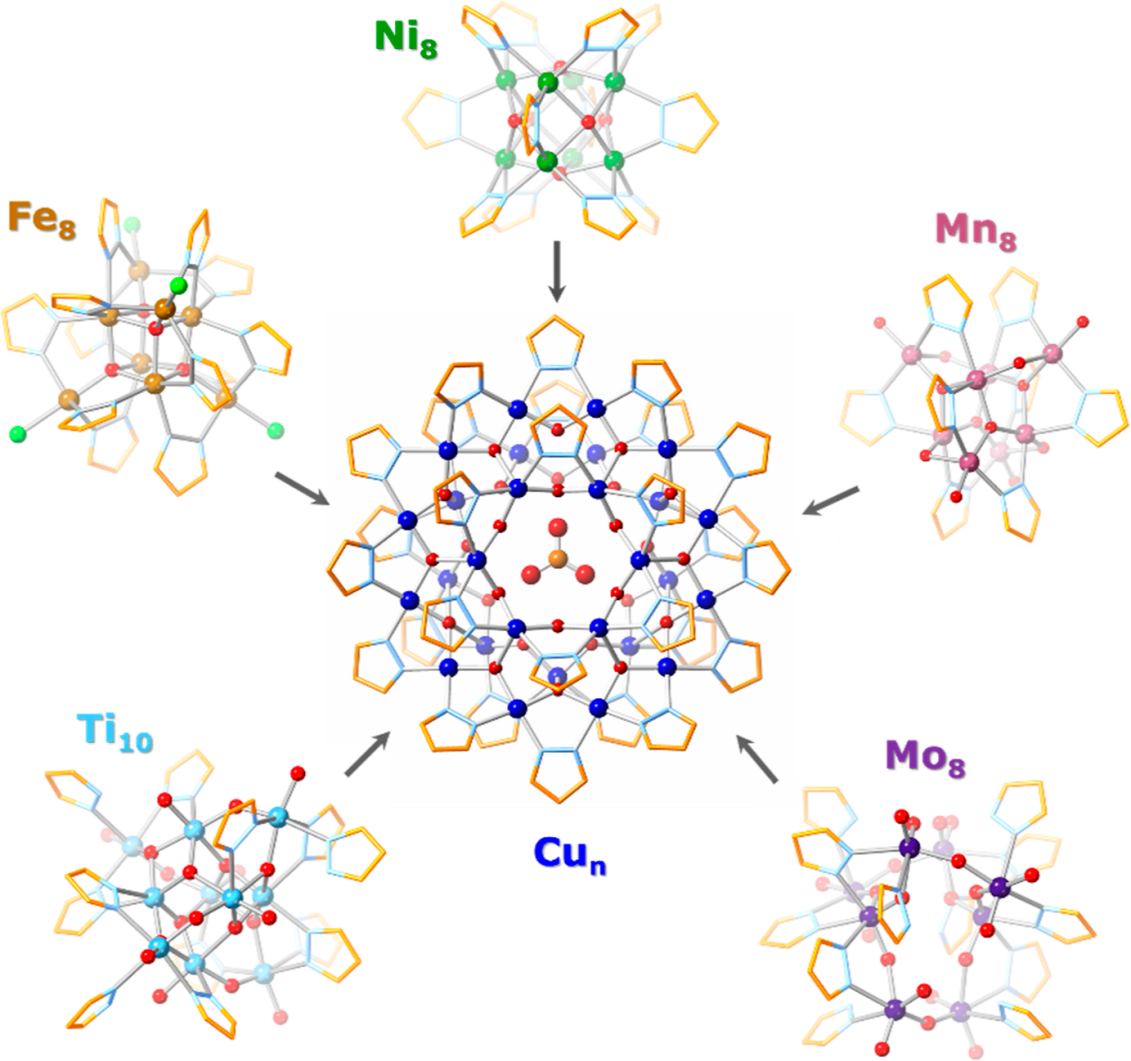
Illustration of the structure of various metal pyrazolate/(hydr)oxide clusters transformed into nanojars in the presence of Cu^2+^ ions.

To further investigate the robustness of the nanojars, we hypothesized that other metal pyrazolate/(hydr)oxide clusters would transform into nanojars in the presence of Cu^2+^ ions if the nanojars are more stable. Thus, we identified five known transition metal clusters that contain pyrazolate and (hydr)oxide ligands and tested their reactivity toward Cu^2+^ ions (Figure 1). In all cases, the metal cluster was transformed into a mixture of (Bu_4_N)_2_[CO_3_⊂{Cu(OH)(pz)}_*n*_] (Cu_*n*_CO_3_; *n* = 27, 29–31) nanojars, which were characterized by electrospray ionization mass spectrometry (ESI-MS) and variable-temperature ^1^H NMR spectroscopy over the 22–150 °C range. Two crystal structures of (MePh_3_P)_2_[CO_3_⊂{Cu(OH)(pz)}_27_], one triclinic (**1**) and one cubic (**2**), were obtained by cation exchange using methyltriphenylphosphonium bromide. The latter displays the highest crystal symmetry obtained to date for a nanojar and is contrasted with solution symmetry obtained from ^1^H NMR spectroscopy. Furthermore, the Cu_*n*_CO_3_ mixture was converted into pure (Bu_4_N)_2_[CO_3_⊂{Cu(OH)(pz)}_27_], and its magnetic properties were studied.

## Results and Discussion

### Synthesis and Mass Spectrometry

**Figure 2:**
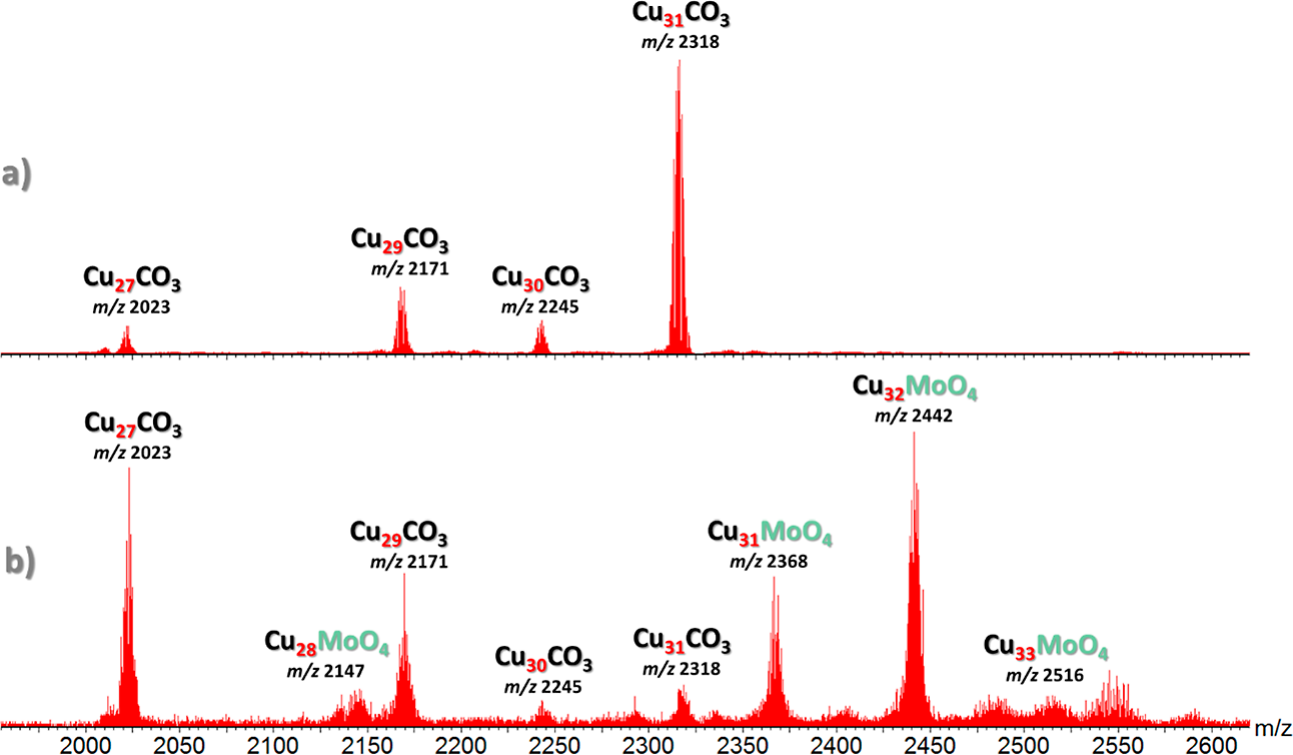
ESI-MS spectra in CH_3_CN of the (a) [CO_3_⊂{Cu(OH)(pz)}_*n*_]^2–^ (Cu_*n*_CO_3_; *n* = 27, 29–31) nanojar mixture obtained from the Ni_8_ cluster and (b) nanojar mixture obtained from the Mo_8_ cluster, which contains [MoO_4_⊂{Cu(OH)(pz)}_*n*_]^2–^ (Cu_*n*_MoO_4_; *n* = 28, 31–33) as well.

Five different metal pyrazolate/(hydr)oxide clusters, namely, (Bu_4_N)_2_[Ni^II^_8_(μ_4_-OH)_6_(μ-pz)_12_],^11^ [Fe^III^_8_(μ_4_-O)_4_(μ-pz)_12_Cl_4_],^12^ [Mn^III^_8_(μ_3_-O)_4_(μ-pz)_8_(μ-OMe)_4_(OMe)_4_],^13^ [Ti^IV^_10_(μ-O)_4_(μ_3_-O)_8_(μ-pz)_8_(pzH)_4_(O^i^Pr)_8_],^14^ and [Mo^VI^_8_O_12_(μ-O)_9_(μ-pz)_6_(pzH)_6_·3pzH·0.5H_2_0],^15^ were each reacted in tetrahydrofuran (THF) with stoichiometric amounts (according to the balanced equations shown in the Experimental Section) of a Cu^2+^ source [Cu(NO_3_)_2_·2.5H_2_O], a CO_3_^2–^ ion source (Na_2_CO_3_·H_2_O or Bu_4_NOH, which traps CO_2_ from the air), and a base (NaOH and/or Bu_4_NOH). The base is needed in order to neutralize the acidity created by hydrated transition metal ions known to break down nanojars into low-nuclearity copper pyrazolate complexes^16^ by conversion to the insoluble (hydr)oxides. During the reaction, the metal pyrazolate/(hydr)oxide cluster completely breaks down and is transformed into nanojars. ESI-MS of the resulting products reveals varying amounts of [CO_3_⊂{Cu(OH)(pz)}_*n*_]^2–^ (Cu_*n*_CO_3_; *n* = 27, *m*/*z* = 2022.91; *n* = 29, *m*/*z* = 2170.54; *n* = 30, *m*/*z* = 2244.61; *n* = 31, *m*/*z* = 2318.16) in CH_3_CN solution (Figures 2 and S1). Unexpectedly, as the nanojars form in the case of the Mo_8_ complex, some of them incorporate molybdate (MoO_4_^2–^) instead of carbonate, giving rise to [MoO_4_⊂{Cu(OH)(pz)}_*n*_]^2–^, indicated by peaks at *m*/*z* 2146.71 (*n* = 28), 2368.15 (*n* = 31), 2441.96 (*n* = 32), and 2515.77 (*n* = 33) (Figure 2b). Analogous peaks were not observed in the case of the Ni, Fe, Mn, and Ti complexes because these metals do not form stable MO_4_^2–^ oxoanions under the given reaction conditions. In the case of the Mo cluster transformation, the Lindqvist hexamolybdate (Bu_4_N)_2_Mo_6_O_19_ was identified as a side-product based on peaks at *m*/*z* 439.84 and 1122.16 in the mass spectrum, corresponding to [Mo_6_O_19_]^2–^ and [(Bu_4_N)Mo_6_O_19_]^−^, respectively (Figure S2).

### Structural Analysis by X-ray Crystallography

**Figure 3:**
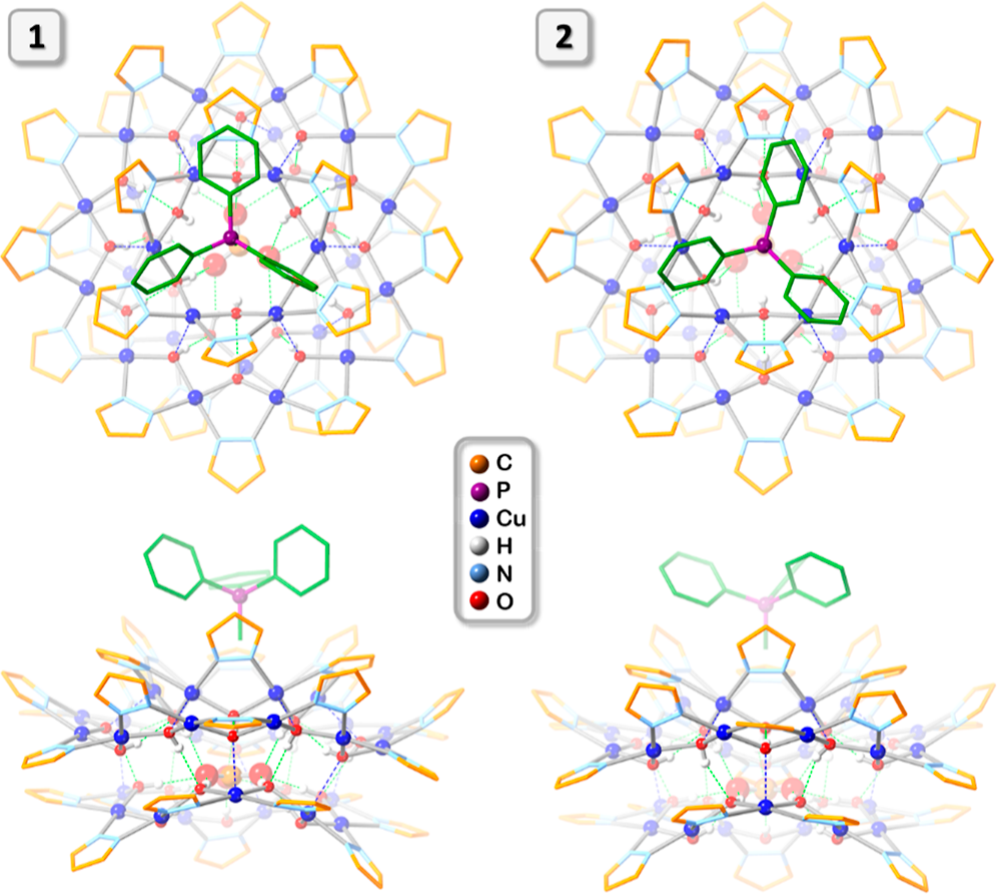
Comparison of the crystal structures of (MePh_3_P)_2_[CO_3_⊂{Cu(OH)(pz)}_27_] in **1** and **2** (top and side-views). Green and blue dotted lines indicate hydrogen bonds and axial Cu···O interactions, respectively. Lattice solvent molecules, C–H bond H atoms, and one of the counterions are omitted for clarity, and only the major component is shown for disordered moieties. C atoms of MePh_3_P^+^ are shown in green.

**Figure 4:**
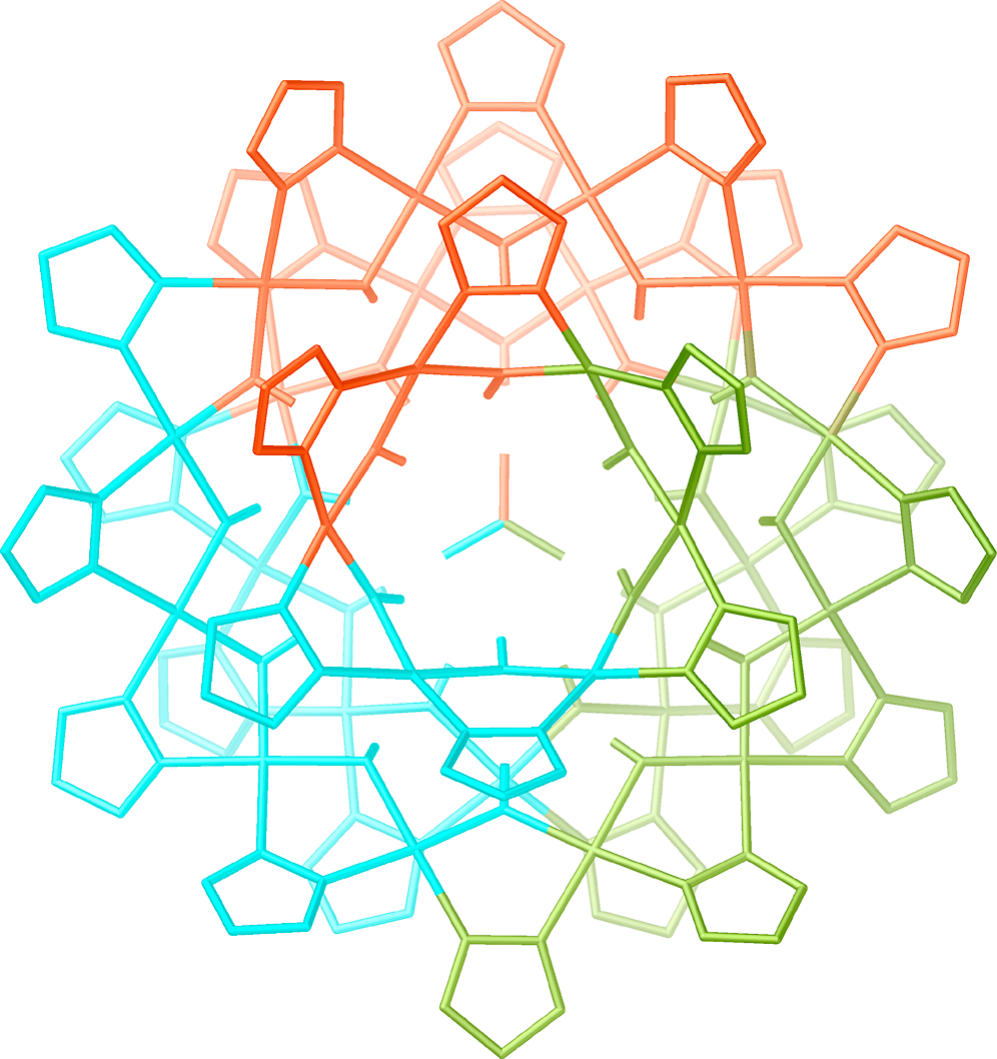
Illustration of the 3-fold symmetry of the nanojar scaffold in **2**.

Two types of crystals (prisms and cubes) were obtained by vapor diffusion of pentane into a *p*-xylene/nitrobenzene solution of Cu_*n*_CO_3_ (Bu_4_N^+^ salt) in the presence of MePh_3_P^+^Br^–^ as an additive, which correspond to two polymorphs of (MePh_3_P)_2_[CO_3_⊂{Cu(OH)(pz)}_27_]: one triclinic, *P*1̅ (**1**), with the nanojar unit on a general position, and the other cubic, *I*23 (**2**), with the nanojar unit centered on a *C*_3_ rotation axis perpendicular to the Cu_*x*_ (*x* = 6, 9, and 12) rings (Table S1 and Figures 3 and 4). Upon crystallization, both original Bu_4_N^+^ counterions were replaced by MePh_3_P^+^ in **1**. In **2**, at least one Bu_4_N^+^ unit (adjacent to the Cu_6_ ring) is replaced by a MePh_3_P^+^. The identity of the second counterion in **2** could not be determined due to extensive disorder with solvent molecules, but it is likely also MePh_3_P^+^ due to the additional aromatic interactions it provides both with pz moieties and with other MePh_3_P^+^ counterions within the crystal lattice. In **1**, the second MePh_3_P^+^ unit (adjacent to the Cu_9_ ring) does interact with a neighboring MePh_3_P^+^ unit across an inversion center, leading to a parallel pair of Ph groups [crystallographically imposed; centroid–centroid distance: 3.667(5) Å, plane-centroid distance: 3.293(6) Å] (Figure S4). In addition to the *C*_3_ rotational symmetry, the nanojar framework in both **1** and **2** is close to being symmetrical relative to three σ mirror planes running along the *C*_3_ axis at 60° from each other.

**Figure 5:**
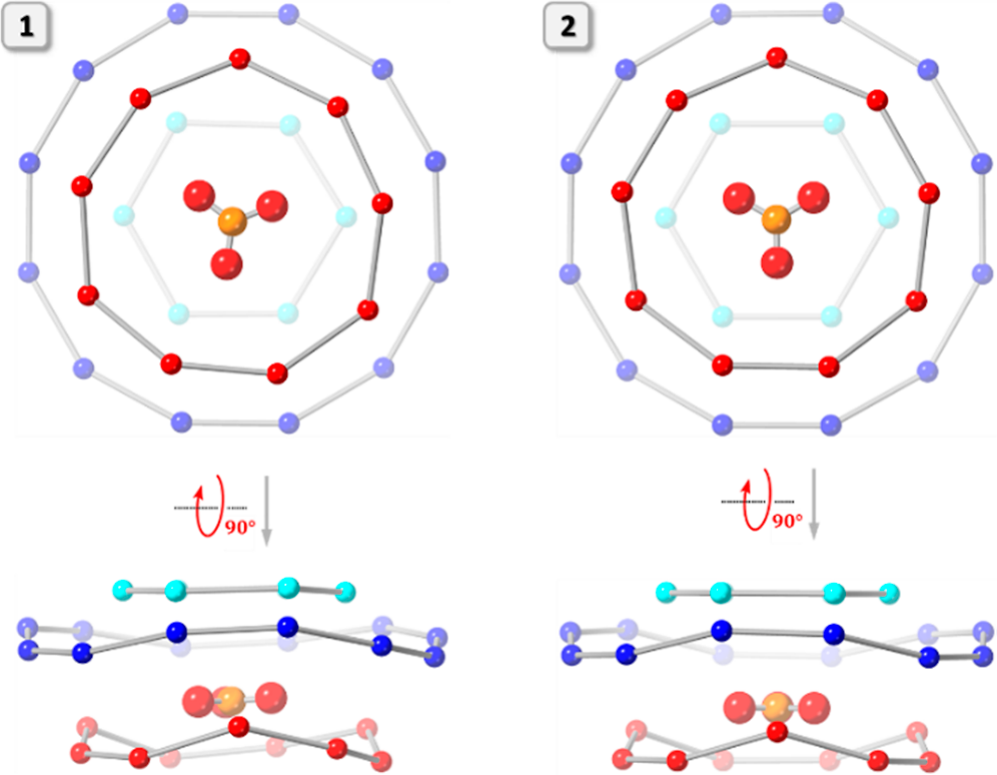
Comparison of the Cu_6_, Cu_12_, and Cu_9_ rings in **1** and **2** (only Cu atoms are shown).

**Figure 6:**
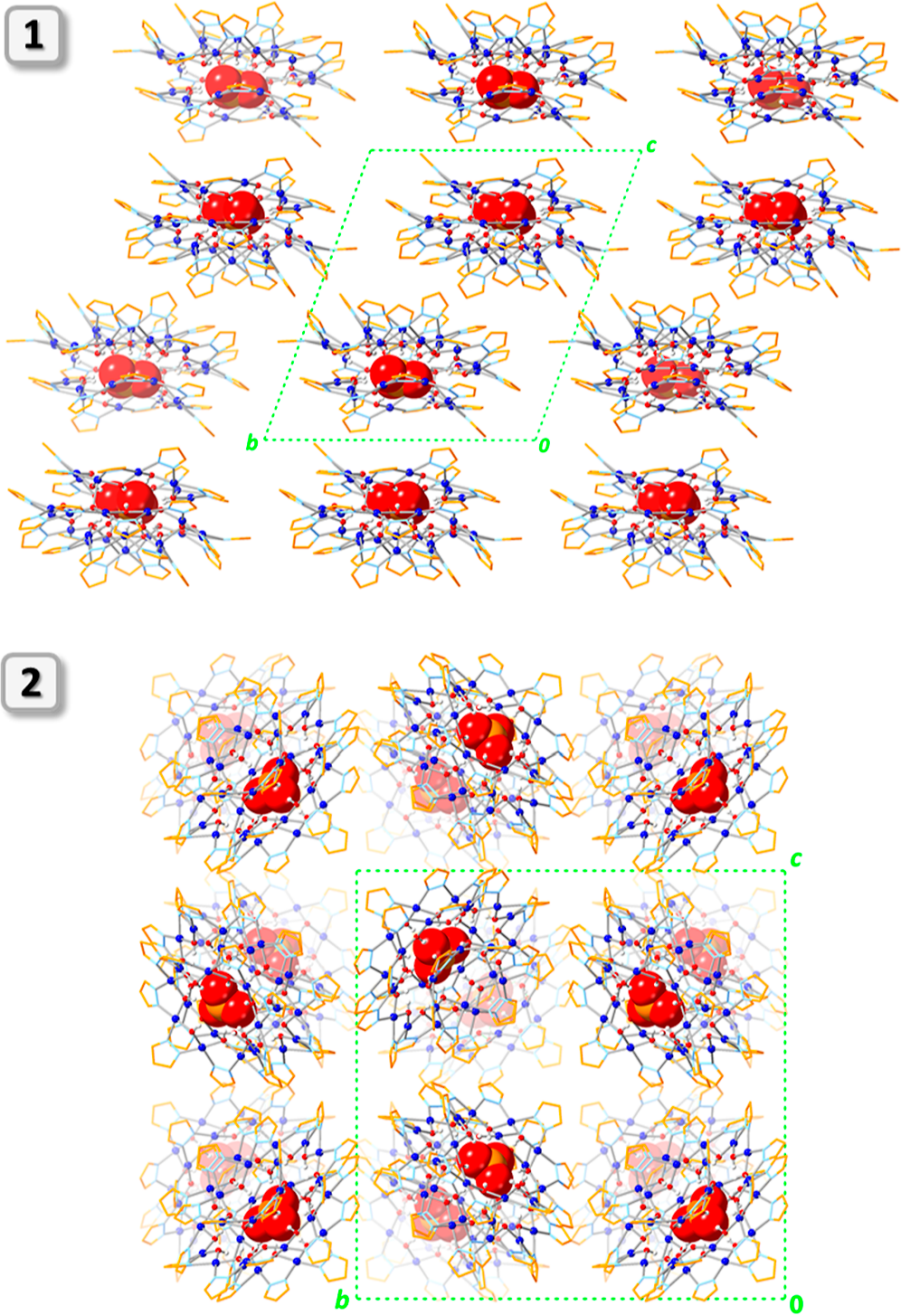
Comparison of the packing diagrams (along the *a* axis) of **1** and **2**. C–H bond H atoms, counterions, and solvent molecules are omitted for clarity, and only the major component is shown for disordered moieties.

The structures of the nanojar frameworks in the two polymorphs **1** and **2** are similar to each other and to the one in a pseudopolymorph of **1** obtained earlier from toluene/cyclohexane^4^ and Bu_4_N^+^ counterions, with slight differences observed in the geometry of the Cu_*x*_ rings and the orientation of the CO_3_^2–^ ion within the nanojar cavity (Figures 3 and 5, Tables S2–S9). These differences, as well as the loss of the *C*_3_ symmetry in **1**, appear to be caused by the orientation of the MePh_3_P^+^ counterions relative to the nanojar. In both **1** and **2**, one MePh_3_P^+^ counterion is positioned close to the Cu_6_ ring. As seen in Figure 3, however, the three phenyl groups of MePh_3_P^+^ are arranged symmetrically above the Cu_6_ ring only in **2**. Here, the three equivalent Ph groups form one type of aromatic interaction with one set of the Cu_6_ ring pyrazolate units [Ph···pz dihedral angle: 23.6(4)°; centroid–centroid distance: 4.226(6) Å] and another type with the other, symmetry-independent set of pyrazolate units [Ph···pz dihedral angle: 52.5(4)°; centroid–centroid distance: 4.549(6) Å]. In contrast, in **1**, not only is the position of MePh_3_P^+^ not symmetrical relative to the Cu_6_ ring but also its Ph groups are rotated to different degrees compared to that in **2** (with one Ph group disordered over two positions with 0.68/0.32 occupancy). Consequently, a different pattern of aromatic interactions arises in compound **1**. The overall packing of nanojars in the crystal lattice is also very different in **1** compared to that in **2** (Figure 6). A less compact packing is observed in the case of the cubic symmetry lattice of **2**, which displays large interstitial voids, also illustrated by the lower corresponding calculated crystal density of 1.395 vs 1.683 g·cm^–3^ in the case of **1**.

The structural parameters (Cu–O and Cu–N bond lengths, *trans* and *cis* N–Cu–O angles, and Cu···Cu distances) within the Cu_*n*_-rings of **1** and **2** are practically identical (Table S2). The positions of the Cu_9_ ring relative to that of the Cu_12_ ring and the bonding between these two rings in **1** and **2**, however, are different. In **2**, there are only three axial Cu···O interactions [2.411(4) Å, symmetry related] between the Cu_9_ and Cu_12_ rings, spanning less than the sum of the van der Waals radii of Cu and O (2.92 Å), whereas in **1**, there are five such interactions [2.396(4)–2.815(4) Å, average: 2.562(4) Å]. Nevertheless, the hydrogen bonding pattern between the Cu_9_ and Cu_12_ rings [12 bonds averaging 2.781(5) Å in **1** and 2.797(6) Å in **2]** is again similar (Table S2). Perhaps the most significant difference between **1** and **2** is the position of the incarcerated CO_3_^2–^ ion, which is centered in a well-defined position within the nanojar cavity in **2** [12H bonds to the Cu_6_ and Cu_9_ rings shorter than 3.2 Å, averaging 2.838(6) Å] but is disordered over two positions (0.82/0.18 occupancy) in **1** [for the major unit: 13H bonds to the Cu_6_ and Cu_9_ rings shorter than 3.2 Å, averaging 2.880(6) Å].

To compare the overall shapes of the nanojar units in **1** and **2**, the dihedral angles and the component fold and twist angles between the mean planes of pyrazolate moieties and adjacent Cu–O–Cu units (as defined earlier)^17^ were analyzed. As shown in Table S3, the corresponding angle averages for the different Cu_*x*_ rings are rather consistent in **1** and **2**, although some significant differences are observed [notably for the twist angles in the Cu_6_ rings, which vary from 0.91(16) to 10.30(17)° in **1** compared to 0.2(3) to 1.7(2)° in **2**]. Furthermore, the dihedral angles and the component fold and twist angles between the mean planes of adjacent pyrazolate moieties were analyzed (Table S4). The most notable difference is for the twist angles in the Cu_9_ rings, which range from 0.6(3) to 48.0(2)° in **1** compared to 11.2(3) to 49.8(7)° in **2**. In terms of the geometry of the Cu_*x*_ rings in **1** and **2**, significant differences are observed for the deviations of the various Cu atoms from the Cu_*x*_ mean planes (Table S5).

It is noteworthy that the non-centrosymmetric, body-centered cubic space group *I*23 (prototype: Ga_4_Ni)^18^ observed in the case of **2** is very rare: only 232 (0.018%) out of a total of almost 1.3 million structures in the Cambridge Structural Database (CSD) belong to this space group (2.7% of cubic structures, which represent 0.67% of all structures).^19^ The corresponding Schoenflies point group is *T*, with four *C*_3_ and three *C*_2_ axes as the symmetry elements. Within the crystal lattice of **2**, nanojar units are centered on the *C*_3_ axes and in-between the *C*_2_ axes.

### Variable-Temperature ^1^H NMR Characterization

**Figure 7:**
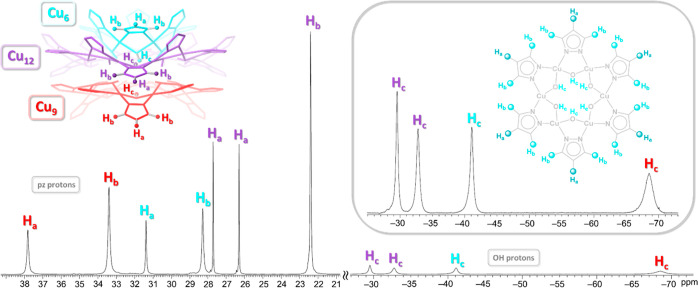
^1^H NMR spectrum in DMSO-*d*_6_ at ambient temperature of pure (Bu_4_N)_2_[CO_3_⊂{Cu(OH)(pz)}_27_]. Inset shows a close-up of the OH region of the spectrum as well as a drawing of the Cu_6_ ring, illustrating the equivalency of the pz and OH groups within Cu_*x*_ rings.

For the NMR studies of carbonate nanojars, a sample of (Bu_4_N)_2_[CO_3_⊂{Cu(OH)(pz)}_*n*_] (*n* = 27, 29–31) was prepared directly from Cu(NO_3_)_2_·2.5H_2_O, Bu_4_NOH, NaOH, and Na_2_CO_3_ in THF.^4^ The distribution of the nanojars of different sizes in this sample is shown in Figure S3. A sample of pure (Bu_4_N)_2_[CO_3_⊂{Cu(OH)(pz)}_27_] (used for the magnetism studies detailed in the next section) was obtained from a THF solution of Cu_*n*_CO_3_ (*n* = 27, 29–31) by precipitation with hexanes. In contrast to the solid-state structure, which allows for *C*_3_ as maximum rotational symmetry in the case of Cu_27_CO_3_ (Figure 4), ^1^H NMR indicates that in solution, all pz moieties as well as OH groups are identical within each Cu_*x*_ ring with the exception of the Cu_12_ ring (Figure 7). For the latter, two sets of signals are observed for both the pz and the OH groups, due to the six of them being oriented toward the Cu_6_ ring, whereas the other six are oriented toward the Cu_9_ ring. Despite the paramagnetism of the Cu^2+^ centers, which causes a drastic downfield shift of the pz proton peaks and an even more drastic upfield shift of the OH proton peaks, as well as a broadening of the peaks and loss of the *J* coupling between nuclei, relatively sharp peaks are observed in the ^1^H NMR spectrum (especially for the pz protons, which are three bonds away from the Cu^2+^ centers, whereas the OH protons are only two bonds away). This suggests strong antiferromagnetic interactions between Cu atoms of the nanojar. Indeed, magnetic susceptibility measurements described in the following section confirm the strong antiferromagnetic coupling in Cu_27_CO_3_. As seen in Figure 7, the extent of this coupling leading to a reduction of spin density is different for the various Cu_*x*_ rings. The odd-membered Cu_9_ ring appears to possess the largest spin density, indicated by the most prominent hyperfine shifts and the fastest relaxation.

**Figure 8:**
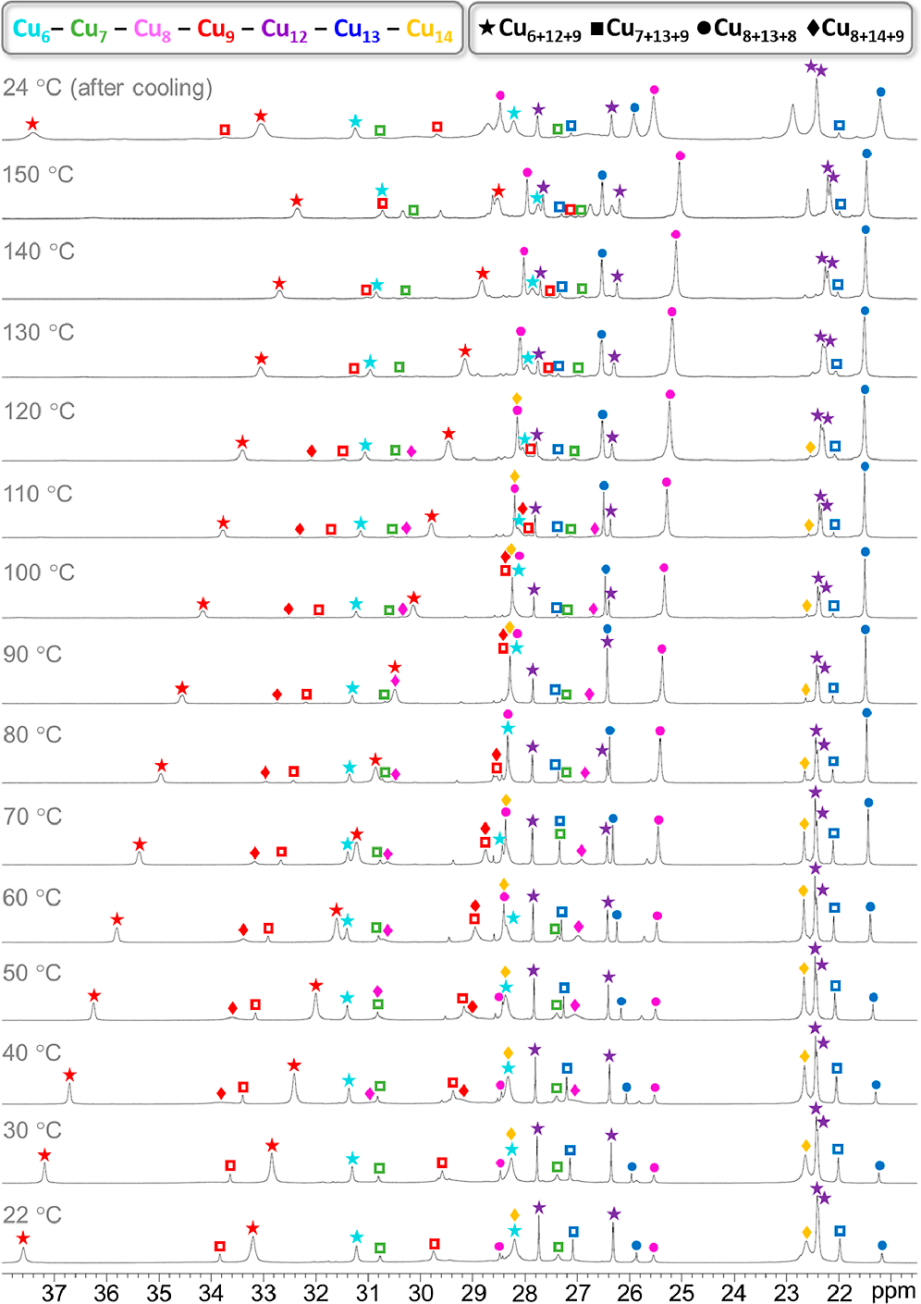
Variable-temperature ^1^H NMR spectra of the Cu_*n*_CO_3_ (*n* = 27, 29–31) nanojar mixture in DMSO-*d*_6_, showing pyrazolate proton signals in the 21–38 ppm window. The temperatures shown are the target temperatures of the probe.

**Figure 9:**
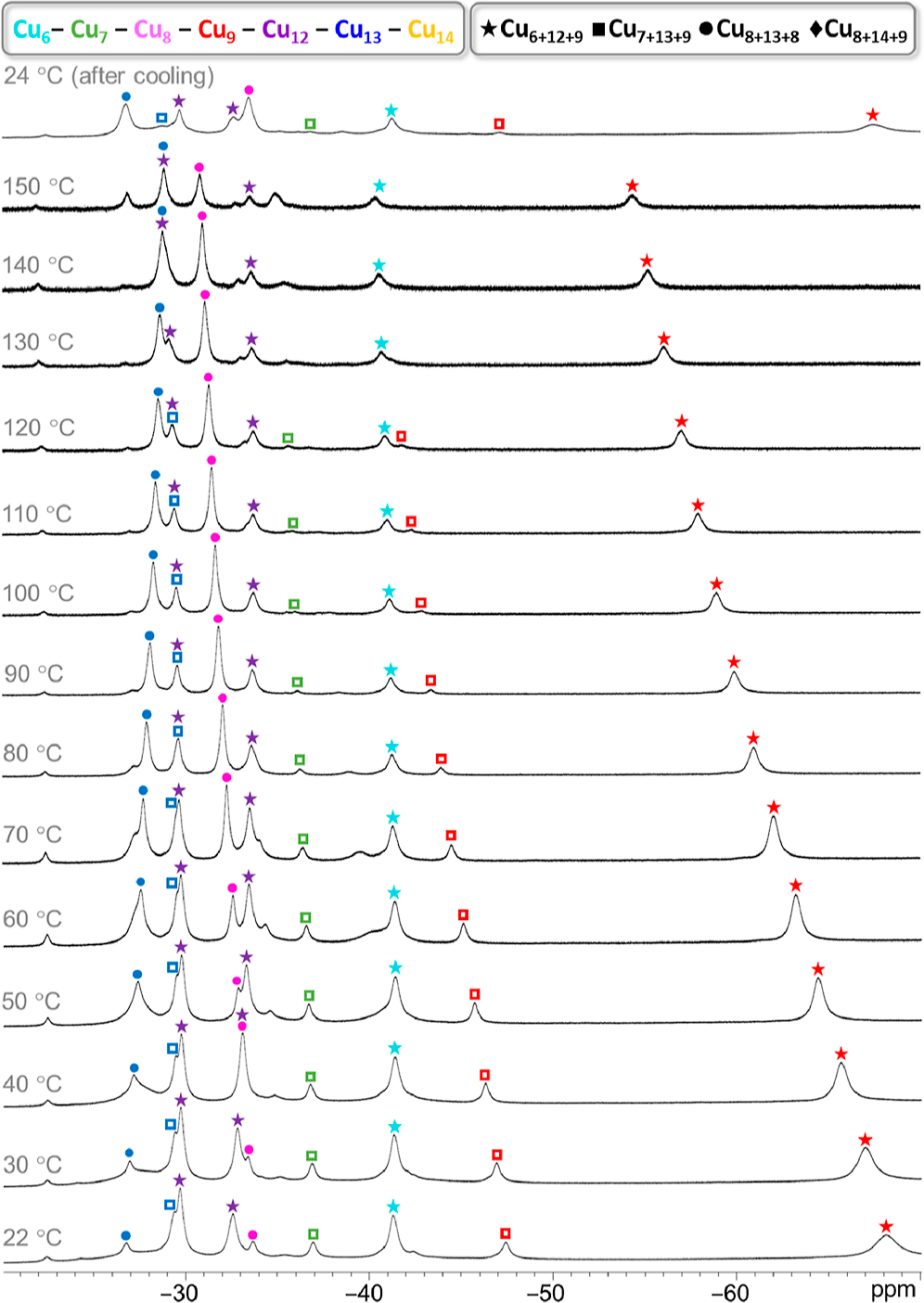
Variable-temperature ^1^H NMR spectra of the Cu_*n*_CO_3_ (*n* = 27, 29–31) nanojar mixture in DMSO-*d*_6_, showing OH proton signals in the (−26)–(−70) ppm window. The temperatures shown are the target temperatures of the probe.

To offer additional data complementing earlier ^1^H NMR characterizations of the Cu_*n*_CO_3_ mixture (*n* = 27, 29–31),^4^ we now present individual chemical shift values for the variable-temperature ^1^H NMR measurements in DMSO-*d*_6_ over the 22–150 °C range (Table S10). The additional spectrum collected at ambient temperature after cooling the sample from 150 °C confirms that the nanojar mixture survives heating to 150 °C in DMSO-*d*_6_ for 1 h without decomposition (Figures 8 and 9). However, the composition of the mixture changes. Initially, the mixture consists of substantial amounts of Cu_27_CO_3_, Cu_29_CO_3_ (Cu_7+13+9_CO_3_, where 7, 13, and 9 indicate the component ring sizes), and Cu_31_CO_3_ and small amounts of Cu_29_CO_3_ (Cu_8+13+8_CO_3_) and Cu_30_CO_3_. Upon heating, the larger nanojars (Cu_31_CO_3_ and Cu_30_CO_3_) gradually decompose and convert to Cu_8+13+8_CO_3_, which becomes the dominant species at 150 °C. The amount of Cu_7+13+9_CO_3_ is also reduced at higher temperatures, although peaks corresponding to this nanojar are still discernible at 150 °C.

The NMR studies of the Cu_*n*_CO_3_ nanojar mixture also reveal that in addition to the coupling between Cu atoms *within* Cu_*x*_ rings, there is coupling *between* Cu_*x*_ rings as well, indicated by the very different chemical shifts of the Cu_9_ ring pz protons in Cu_6+12+9_ (37.63 and 33.24 ppm) compared to that in Cu_7+13+9_ (33.87 and 29.79 ppm) and Cu_8+14+9_ (∼34.2 and ∼29.6 ppm). Similarly, the chemical shifts of the Cu_8_ ring pz protons are different in Cu_8+13+8_ (28.52 and 25.58 ppm) and Cu_8+14+9_ (∼31.0 and ∼27.2 ppm), as are the corresponding values for Cu_13_ in Cu_8+13+8_ (25.91 and 21.21 ppm) and Cu_7+13+9_ (27.12 and 22.02 ppm). An even more drastic difference is observed for the OH protons of the Cu_9_ ring in Cu_6+12+9_ (−68.08 ppm) compared to those in Cu_7+13+9_ (−47.36 ppm) (the corresponding signal for Cu_8+14+9_ is too broad at ambient temperature to be observed). In addition to six H bonds between their OH groups, the Cu_6_ ring is attached to the Cu_12_ ring in Cu_27_CO_3_ by six axial Cu–O bonds ranging from 2.370(4) to 2.477(4) Å [average: 2.429(4) Å] in **1** and from 2.412(4) to 2.435(4) Å [average: 2.424(4) Å] in **2** (Table S2). Thus, the OH groups within the resulting Cu_3_(μ_3_-OH) units can also mediate inter-ring coupling. Within these Cu_3_(μ_3_-OH) moieties, the Cu_6_/Cu_12_ intraring Cu···Cu distances vary from 3.164(1) to 3.394(1) Å [average: 3.302(1) Å] in **1** and from 3.153(1) to 3.377(1) Å [average: 3.293(1) Å] in **2**, whereas the shortest inter-ring Cu···Cu distances vary from 3.481(1) to 3.530(1) Å [average: 3.499(1) Å] in **1** and from 3.433(1) to 3.496(1) Å [average: 3.465(1) Å] in **2**. In contrast, there are only five axial Cu_9_/Cu_12_ intraring Cu···O distances shorter than the sum of the van der Waals radii of Cu and O (2.92 Å) in **1** [2.396(4)–2.815(4) Å, average: 2.562(4) Å] and only three in **2** [2.411(4) Å, symmetry related], with the shortest inter-ring Cu···Cu distances in the corresponding Cu_3_(μ_3_-OH) units of 3.513(1)–3.584(1) Å [average: 3.548(1) Å] in **1** and 3.587(1) Å in **2**. The four Cu atoms of the Cu_9_ ring in **1** (and six in **2**) that are not part of Cu_3_(μ_3_-OH) units can still interact with the Cu_12_ ring through space as some of them are found at comparable Cu···Cu distances of 3.525(1) and 3.527(1) Å in **1** and **2**, respectively. Indeed, there must be significant coupling between the Cu_9_ and Cu_12_ (or Cu_13_/Cu_14_) rings as well, as documented by the different chemical shifts of the Cu_9_ ring protons in Cu_6+12+9_, Cu_7+13+9_, and Cu_8+14+9_. It is possible that this inter-ring coupling is also responsible for the reduction of spin density on the odd-membered Cu_*x*_ rings (*x* = 7, 9, and 13), making the corresponding ^1^H NMR resonances observable, with the concomitant increase of spin density on the adjacent even-membered Cu_*x*_ rings (*x* = 6, 8, 12, and 14) which otherwise would display even sharper signals (having no uncompensated spins).

**Figure 10:**
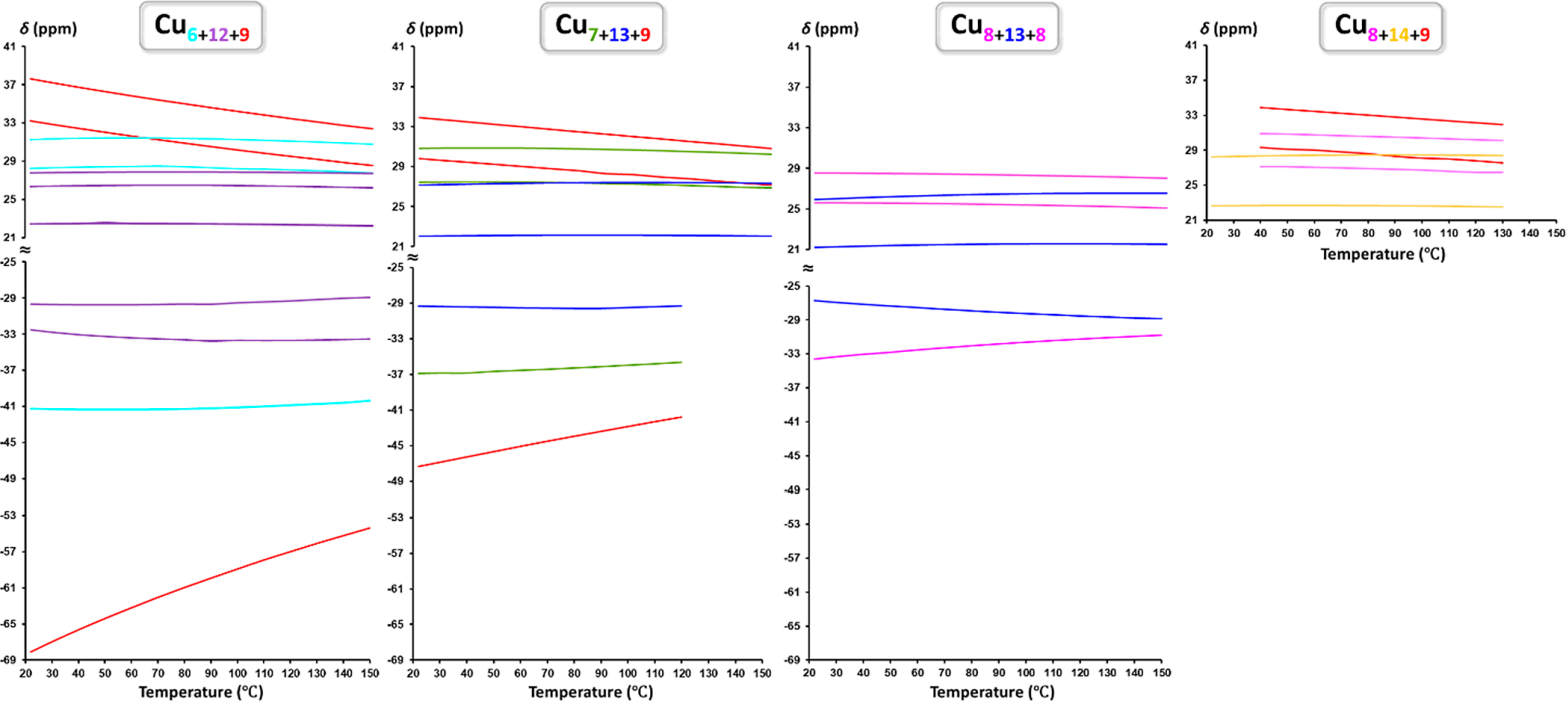
Temperature-dependent variation of the chemical shifts (δ) of different Cu_*x*_ ring protons in various Cu_*n*_CO_3_ nanojars in DMSO-*d*_6_.

Variable-temperature NMR experiments conducted on Cu_*n*_CO_3_ (*n* = 27, 29–31) indicate different temperature dependence of the chemical shifts (δ) for different Cu_*x*_ rings (Figure 10). The peaks corresponding to the Cu_9_ (and to a lesser extent Cu_7_ and Cu_8_) rings display large variations with temperature, becoming less paramagnetically shifted at higher temperatures, which indicates Curie behavior (δ ∝ 1/*T*). The δ values of all other Cu_*x*_ rings show only slight variations with temperature, except for the OH signal of the Cu_13_ ring in Cu_7+13+9_, which shows anti-Curie behavior becoming more paramagnetically shifted at higher temperatures. This last observation, which suggests transfer of spin density from one Cu_*x*_ ring to another at higher temperatures, along with the fact that the δ values of Cu_9_ rings in different nanojars display different temperature dependences (for example, the OH signal of the Cu_9_ ring spans ∼14 ppm in Cu_6+12+9_ but only ∼7 ppm in Cu_7+13+9_ in the 22–150 °C range) as well as different hyperfine shifts, is further evidence for the magnetic coupling *between* Cu_*x*_ rings in nanojars. The corresponding Curie plots of the ^1^H NMR data, obtained by plotting δ as a function of 10^3^/T, are shown in Figure S7.

### UV–Vis Spectroscopy

The UV–vis spectrum of Cu_27_CO_3_ in THF (Figure S8) displays two peaks with absorption maxima at 345 and 601 nm, attributed to charge transfer and *d*–*d* transitions, respectively, with extinction coefficients of ε_345 nm_ = 2.3 × 10^4^ L mol^–1^ cm^–1^ and ε_601 nm_ = 2.2 × 10^3^ L mol^–1^ cm^–1^, respectively. The corresponding values for the Cu_*n*_CO_3_ mixture (*n* = 27, 29–31) are ε_350 nm_ = 2.9 × 10^4^ L mol^–1^ cm^–1^ and ε_599 nm_ = 2.4 × 10^3^ L mol^–1^ cm^–1^, respectively.

### Thermogravimetric Analysis

Solid (Bu_4_N)_2_[CO_3_⊂{Cu(OH)(pz)}_27_] is stable on heating under *N*_2_(*g*) up to ∼205 °C, when loss of (Bu_4_N)_2_CO_3_ begins (calc. 12%, obs. 12%) (Figure S9). At ∼ 230 °C, the resulting [Cu(OH)(pz)]_∞_ breaks down and loss of pyrazole takes place (pz^–^ + HO^–^ → pzH + O^2–^), along with the reduction of the CuO decomposition product to Cu_2_O by ∼300 °C (total loss: calc. 57%, obs. 57%).

### Magnetic Properties

**Figure 11:**
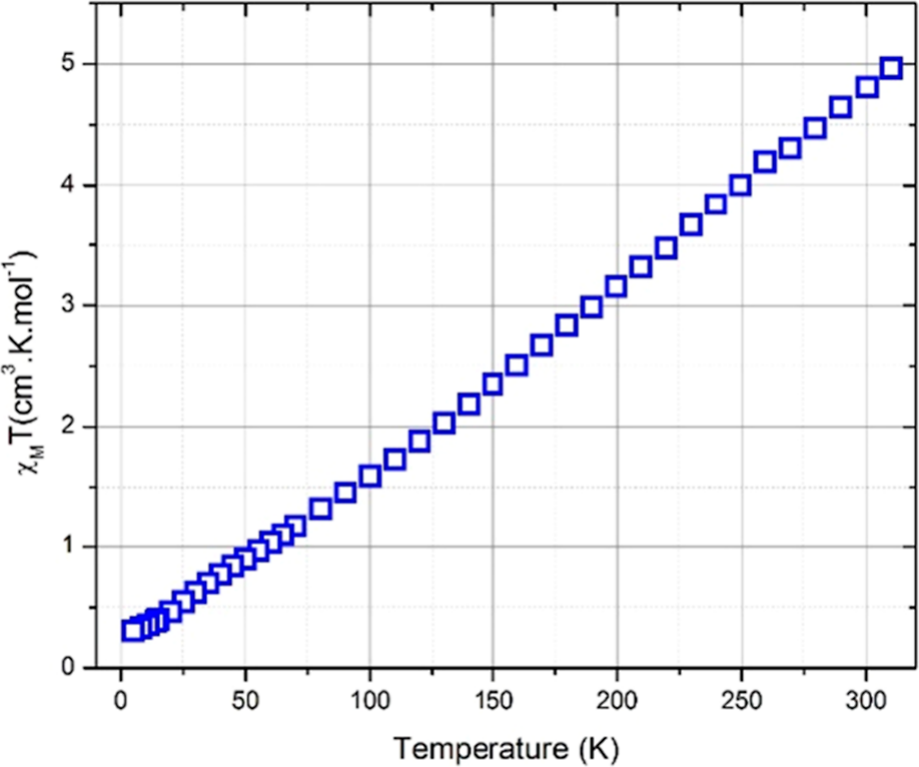
Temperature dependence of χ_M_*T* for (Bu_4_N)_2_[CO_3_⊂{Cu(OH)(pz)}_27_] measured at *B* = 0.1 T (1000 Oe).

**Figure 12:**
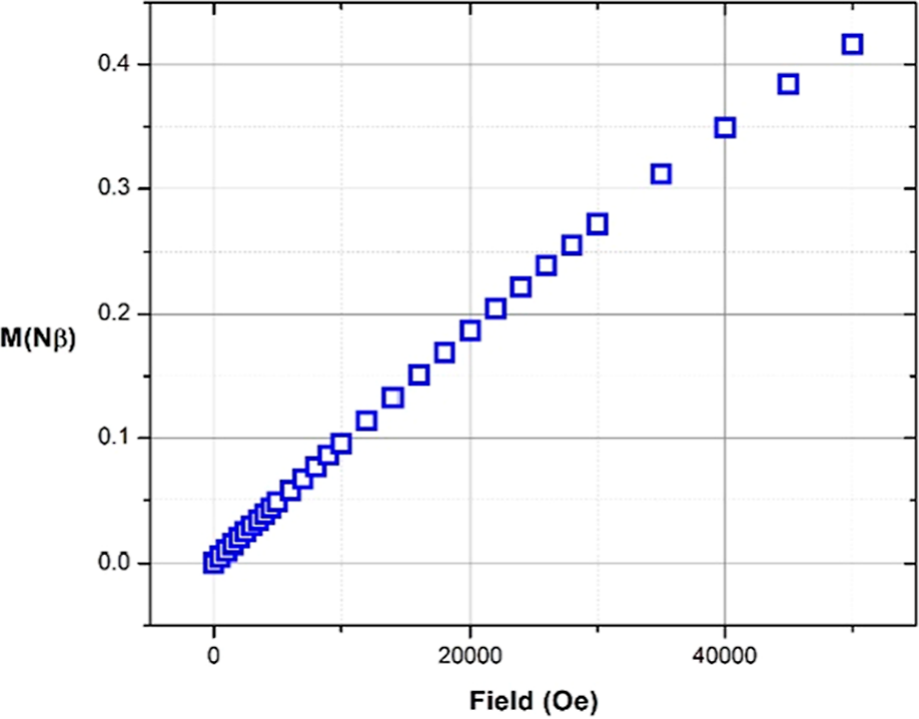
Field dependence of the magnetization of (Bu_4_N)_2_[CO_3_⊂{Cu(OH)(pz)}_27_] measured at 5 K.

The DC magnetic susceptibility data for (Bu_4_N)_2_[CO_3_⊂{Cu(OH)(pz)}_27_] are shown as χ_M_*T* vs *T* from 310 to 1.8 K (under an external field of 0.1 T) in Figure 11. The χ_M_*T* value at 300 K is about 4.8 cm^3^ mol^–1^ K, which corresponds to an effective magnetic moment of 6.2 μ_B_. This value is significantly lower than the theoretical value of μ_eff_ = 9.0 calculated for 27 noninteracting Cu^2+^ ions (assuming *g* = 2). The χ_M_*T* value steadily decreases as the temperature drops and finally reaches a value of 0.37 cm^3^ mol^–1^ K at 1.8 K (μ_eff_ = 1.7), which suggests a possible *S* = 1/2 ground state. Thus, the magnetic susceptibility measurements indicate strong antiferromagnetic coupling between the Cu^2+^ ions, which cannot be overcome by thermal perturbation, even at room temperature. Both pyrazolate and O(H) ligands are known to mediate such coupling.^20^ The field-dependent magnetization increases from 0 to 5 T due to the field-induced population of states with higher spins (Figure 12). The close to linear increase in magnetization without saturation (corresponding to an *S* = 1/2 spin ground state) at high magnetic fields suggests spin frustration (competing antiferromagnetic interactions that cannot be simultaneously satisfied). Similar magnetic behavior was observed in a Cu^II^_27_ cluster based on *o*-vanillinoxime,^21^ as well as in Cu^II^_12_ and Cu^II^_20_ keplerates.^22^ For [CO_3_⊂{Cu(OH)(pz)}_27_]^2–^, both an *S* = 1/2 ground state and spin frustration can be rationalized based on its structure comprising three Cu_*x*_ rings (*x* = 6, 9, and 12). Thus, in the even-membered Cu_6_ and Cu_12_ rings, the antiparallel spins on consecutive Cu centers [Cu···Cu distances of 3.2052(9)–3.3942(11) and 3.2030(8)–3.3722(10) Å, respectively, based on the structure of **1**] are all compensated, whereas in the odd-membered Cu_9_ ring [Cu···Cu distances of 3.1638(10)–3.3624(8) Å], one of the spins remains uncompensated.

## Conclusions

In summary, we demonstrated the superior robustness of the nanojar framework compared to other metal pyrazolate/(hydr)oxide complexes by transforming Ni^II^_8_, Fe^III^_8_, Mn^III^_8_, Ti^IV^_10_, and Mo^VI^_8_ clusters into [CO_3_⊂{Cu(OH)(pz)}_*n*_]^2–^ (*n* = 27, 29–31) in the presence of Cu^2+^ ions in THF solution. The resulting nanojars were characterized in CH_3_CN and DMSO-*d*_6_ solutions by ESI-MS and variable-temperature ^1^H NMR spectroscopy over the 22–150 °C range, respectively. The latter shows time-averaged *C*_*n*_ symmetry for the various Cu_*x*_ rings in solution (except for the Cu_12_ ring, which displays two sets of signals), in contrast with the highest possible *C*_3_ symmetry in the solid state observed for the first time in a cubic (*I*23) crystal of (MePh_3_P)_2_[CO_3_⊂{Cu(OH)(pz)}_6+12+9_]. A more compact packing of nanojar units is observed in this cubic crystal lattice than in its lower symmetry (triclinic, *P*1̅) polymorph. As suggested by the NMR experiments in solution, strong antiferromagnetic coupling between the paramagnetic Cu^2+^ centers is observed in the case of (Bu_4_N)_2_[CO_3_⊂{Cu(OH)(pz)}_27_] in the solid state, as well. The magnetic measurements also confirm spin frustration due to the unpaired electron in the Cu_9_ ring. The serendipitous formation of molybdate-incarcerating nanojars, [MoO_4_⊂{Cu(μ-OH)(μ-pz)}_*n*_]^2–^ (*n* = 28, 31–33) from the [Mo^VI^_8_O_12_(μ-O)_9_(μ-pz)_6_(pzH)_6_·3pzH] cluster motivates further studies on the incarceration of even larger oxoanions by nanojars, which are currently underway.

## Experimental Section

### General

All of the commercially available chemicals were used as received. Deionized water was freshly boiled and cooled to room temperature under *N*_2_(*g*). (Bu_4_N)_2_[Ni^II^_8_(μ_4_-OH)_6_(μ-pz)_12_],^11^ [Fe^III^_8_(μ_4_-O)_4_(μ-pz)_12_Cl_4_],^12^ [Mn^III^_8_(μ_3_-O)_4_(μ-pz)_8_(μ-OMe)_4_(OMe)_4_],^13^ [Ti^IV^_10_(μ-O)_4_(μ_3_-O)_8_(μ-pz)_8_(pzH)_4_(O^i^Pr)_8_],^14^ and [Mo^VI^_8_O_12_(μ-O)_9_(μ-pz)_6_(pzH)_6_·3pzH·0.5H_2_0]^15^ were prepared according to the published procedures. NMR, UV–vis, and thermogravimetric analysis (TGA) spectra were collected on a Jeol JNM-ECZS (400 MHz), a Shimadzu UV-1650PC, and a thermal analyzer TGA Q500 instrument (heated at 5 °C/min from 20 to 500 °C under N_2_), respectively.

### Conversion of Metal Pyrazolate/(Hydr)oxide Clusters into Nanojars

*Method* (*a*). The metal cluster (25 μmol) was stirred together with stoichiometric amounts (according to the balanced equations shown below, with an average *n* = 29) of Cu(NO_3_)_2_·2.5H_2_O, Bu_4_NOH (55% in H_2_O; counterion source), NaOH (base), and Na_2_CO_3_·H_2_O (carbonate source) in THF (10 mL) for 3 days in a closed vessel. The reaction mixture was then filtered into water (100 mL) under stirring, and the resulting blue precipitate, identified as (Bu_4_N)_2_[CO_3_⊂{Cu(OH)(pz)}_*n*_] (*n* = 27, 29–31) by ESI-MS, was isolated by filtration and was dried under vacuum. *Method* (*b*). The reactions were carried out similarly by stirring THF solutions in the air, using only Bu_4_NOH as the base (which also provides carbonate ions by reacting with atmospheric CO_2_), according to the balanced equations shown below (with an average *n* = 29).
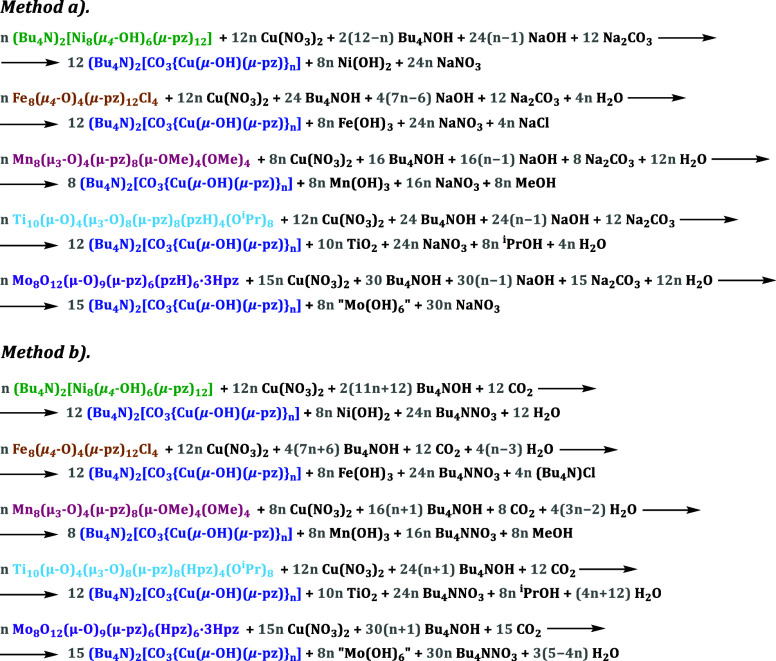


### Synthesis of (Bu_4_N)_2_[CO_3_⊂{Cu(OH)(pz)}_27_]

Cu(NO_3_)_2_·2.5H_2_O (5.0000 g, 21.50 mmol) and pyrazole (1.4635 g, 21.50 mmol) were dissolved in 100 mL of THF in a 200 mL round-bottom flask. Then, Na_2_CO_3_·H_2_O (2.6658 g, 21.50 mmol), NaOH (1.6608 g, 41.52 mmol), and tetrabutylammonium hydroxide (55% in H_2_O, 0.7008 g, 1.48 mmol) were added, and the reaction mixture was stirred in a stoppered flask at room temperature for 3 days. Then, the reaction mixture was filtered and added to hexanes (500 mL) under stirring. The blue precipitate was filtered out and rinsed with hexanes. Yield: 0.7501 g. The purity of the sample was confirmed by ESI-MS (Figure S3) and ^1^H NMR (Figure 7).

### Mass Spectrometry

Mass spectrometric analysis of the nanojars was performed with a Waters Synapt G1 HDMS instrument using electrospray ionization (ESI). 10^–4^–10^–5^ M solutions were prepared in CH_3_CN using either solids or aliquots taken from solutions. Samples were infused by a syringe pump at 5 μL/min, and nitrogen was supplied as the nebulizing gas at 500 L/h. The electrospray capillary voltage was set to −2.5 or +2.5 kV, respectively, with a desolvation temperature of 110 °C. The sampling and extraction cones were maintained at 40 and 4.0 V, respectively, at 80 °C. Reported *m*/*z* values represent averages of the observed isotopic distributions.

### Magnetism

Magnetic data were collected on a Quantum Design MPMS-XL SQUID magnetometer equipped with a 7 T magnet. DC magnetic susceptibility measurements were carried out under a 1000 Oe magnetic field from 310 to 1.8 K, and magnetization measurements were operated at 5 K with an external field from 0 to 5 T. The polycrystalline sample used for the measurements was embedded in solid eicosane to prevent torqueing. Pascal’s constants were used to determine the magnetic correction, which was subtracted from experimental data to obtain the molar susceptibility.

### X-ray Crystallography

Single crystals of **1** and **2** were grown at room temperature by vapor diffusion of pentane into a nitrobenzene/*p*-xylene solution of Cu_*n*_CO_3_ (*n* = 27, 29–31) in the presence of MePh_3_PBr. Once removed from the mother liquor, the crystals are extremely sensitive to solvent loss under ambient conditions and were quickly mounted under a cryostream (150 K) to prevent decomposition. X-ray diffraction data were collected from a single crystal mounted atop a MiTeGen micromesh mount under Fomblin oil with a Bruker AXS D8 Quest diffractometer equipped with a Photon II charge-integrating pixel array detector (CPAD) using graphite-monochromated Mo-K_α_ (λ = 0.71073 Å) radiation. The data were collected using APEX4,^23^ integrated using SAINT,^24^ and scaled and corrected for absorption and other effects using SADABS.^25^ The structures were solved by employing direct methods using ShelXS^26^ or ShelXT^27^ and refined by full-matrix least-squares on *F*^2^ using ShelXL.^28^ C–H hydrogen atoms were placed in idealized positions and refined by using the riding model. Further refinement details and thermal ellipsoid plots (Figures S5 and S6) are provided in the Supporting Information.

## Supporting Information Available

Additional data for MS, X-ray crystallography, ^1^H NMR and UV–vis spectroscopy, and TGA (PDF)
