# Supplementary material for: Conversion of Metal Pyrazolate/(Hydr)oxide Clusters into Nanojars: Solution vs Solid-State Structure and Magnetism
Source: Inorg Chem. 2024 Jun 14;63(26):12290–8. doi: 10.1021/acs.inorgchem.4c01698 (PMC11220756; doi:10.1021/acs.inorgchem.4c01698)
Supplement: Supplementary file 1 — ic4c01698_si_001.pdf [file ic4c01698_si_001.pdf]

Supporting Information for

Conversion of Metal Pyrazolate/(Hydr)oxide Clusters into Nanojars: Solution vs. Solid State Structure and Magnetism

Pooja Singh,[a] Wisam A. Al Isawi,[a] Matthias Zeller[b] and Gellert Mezei[a*]

[a] *Department of Chemistry, Western Michigan University, Kalamazoo, Michigan 49008, USA*

[b] *Department of Chemistry, Purdue University, West Lafayette, Indiana 47907, USA*

[*] Corresponding author. Email: gellert.mezei@wmich.edu

CONTENTS **PAGE**

1. Mass spectrometric data (Figures S1−S3) S2–S3
2. X-ray crystallographic data and refinement details (Figures S4−S6 and Tables S1−S9) S3–S13
3. NMR spectroscopic data (Table S10, Figure S7) S14–S15
4. UV-vis spectroscopic data (Figure S8) S16
5. Thermogravimetric analysis data (Figure S9) S16
6. References S17

1. MASS SPECTROMETRIC DATA

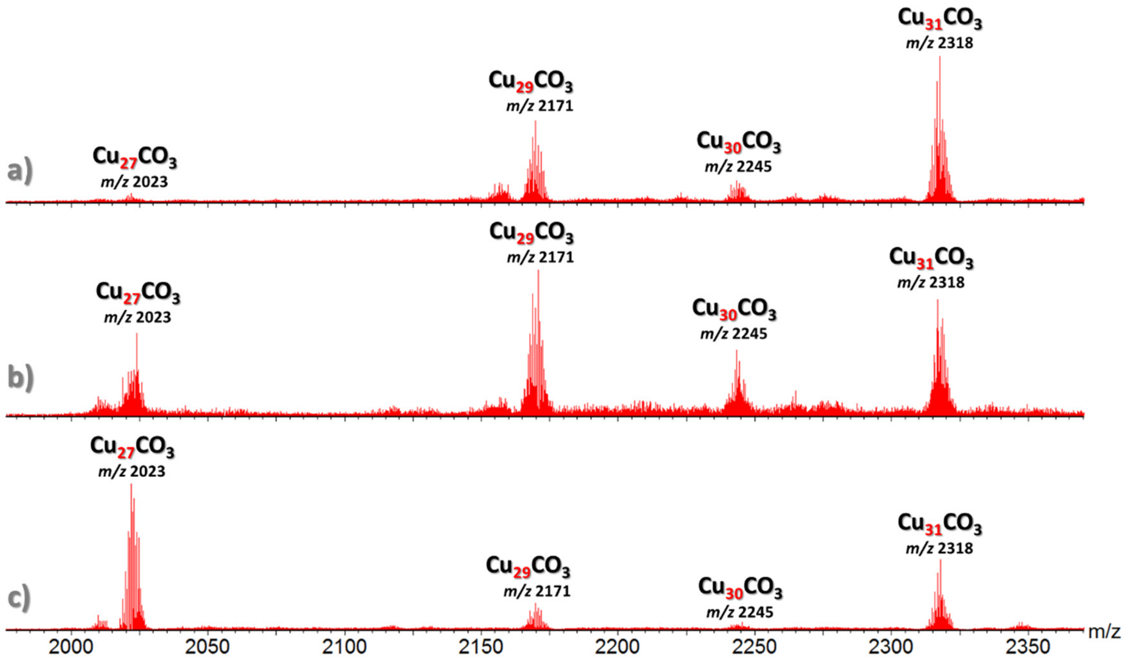

Figure S1. ESI-MS spectra in CH_3CN of the nanojar mixture $(Bu_4N)_2[CO_3\subset\{Cu(OH)(pz)\}_n]$ (Cu_nCO_3; n = 27, 29–31) obtained from a) $[Ti^{IV}_{10}(\mu\text{-}O)_4(\mu_3\text{-}O)_8(\mu\text{-}pz)_8(pzH)_4(O^iPr)_8]$, b) $[Mn^{III}_8(\mu_3\text{-}O)_4(\mu\text{-}pz)_8(\mu\text{-}OMe)_4(OMe)_4]$ and c) $[Fe^{III}_8(\mu_4\text{-}O)_4(\mu\text{-}pz)_{12}Cl_4]$.

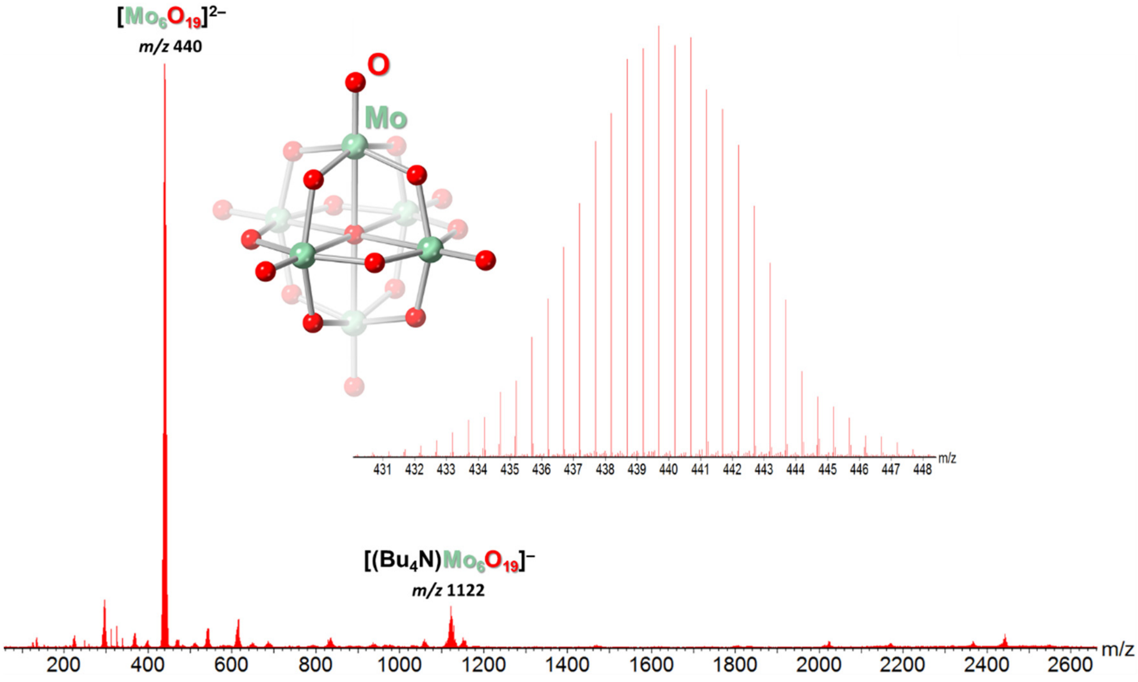

Figure S2. ESI-MS spectrum in CH_3CN of the soluble part of the side-product obtained from the reaction of $Mo^{VI}_8O_{12}(\mu\text{-}O)_9(\mu\text{-}pz)_6(pzH)_6\cdot 3pzH$ with $Cu(OH)_2$ (inset shows the isotopic pattern of the $[Mo_6O_{19}]^{2-}$ ion).

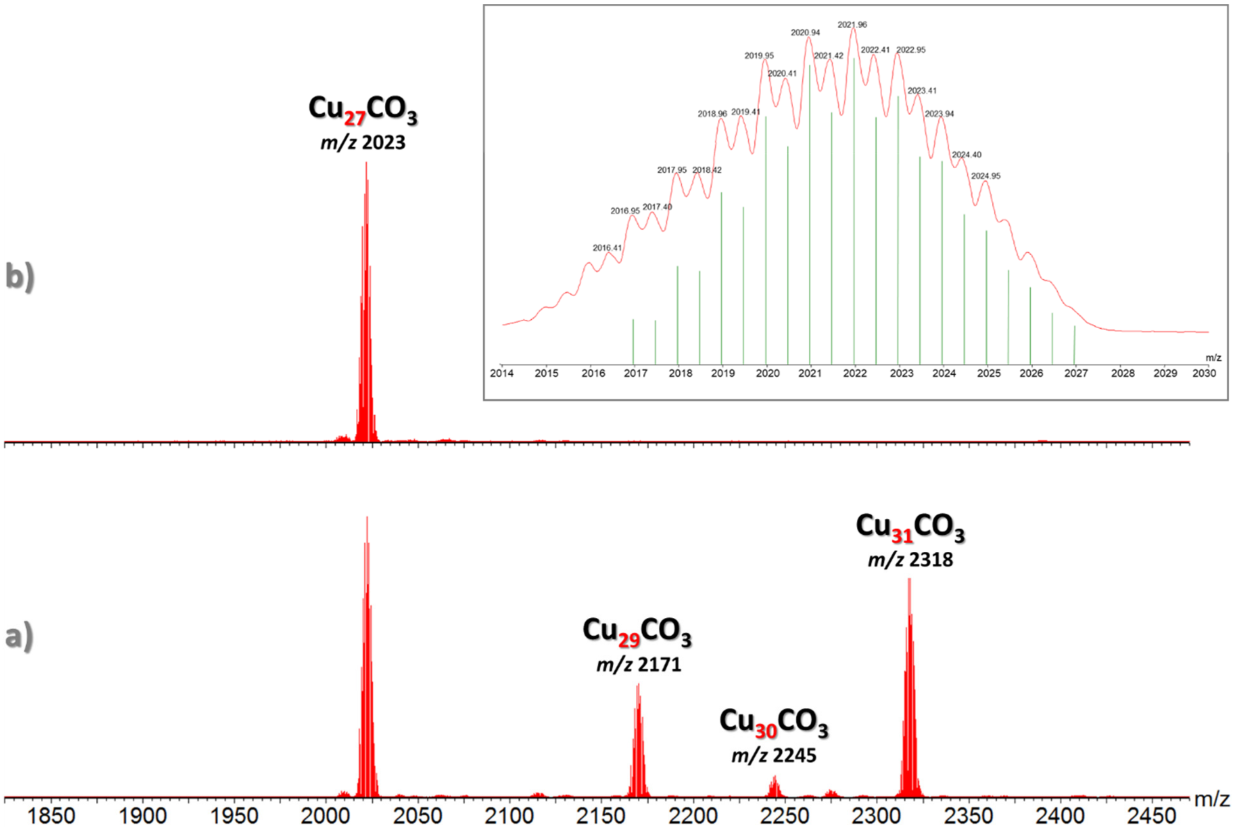

Figure S3. ESI-MS spectra in CH_3CN of a) the nanojar mixture $(Bu_4N)_2[CO_3\subset\{Cu(OH)(pz)\}_n]$ (n = 27, 29–31) used for the VT-NMR experiments and b) the pure $(Bu_4N)_2[CO_3\subset\{Cu(OH)(pz)\}_{27}]$ nanojar, along with its predicted (green) and observed (red) isotopic patterns (inset).

2. X-RAY CRYSTALLOGRAPHIC DATA

1: The carbonate oxygen atoms were refined as two-fold disordered. The disordered moieties were restrained to have similar geometries. U_{ij} components of ADPs for disordered atoms closer to each other than 2.0 Å were restrained to be similar. Subject to these conditions the occupancy ratio refined to 0.822(6)/0.1782(6).

Two pyrazole ligands (of N33 and N41) were refined as disordered. The disordered moieties were restrained to be close to planar and to have similar geometries as another better-defined pyrazole ligand. Cu–N distances for the second disordered pyrazole were restrained to be similar to each other. U_{ij} components of ADPs for disordered atoms closer to each other than 2.0 Å were restrained to be similar. Subject to these conditions the occupancy ratios refined to 0.50(2)/0.50(2) and 0.430(16)/0.570(16).

Hydroxyl H atom positions were refined and O–H distances were restrained to a target value of 0.84(2) Å. The position of H atoms H8O, H12O, H24O and H27O were further restrained based on hydrogen bonding considerations (2.00(2) Å to O1, 1.95(2) Å to O3, 2.00(2) Å to O24 and 2.10(2) Å to O30B, respectively).

One phenyl ring of one countercation was refined as disordered (C109 to C114). The moieties were restrained to have similar geometries as another better-defined fragment of the same kind. U_{ij} components of ADPs for disordered atoms closer to each other than 2.0 Å were restrained to be similar. Subject to these conditions the occupancy ratio refined to 0.631(18)/0.369(18) for C103 through C105, and to 0.323(7)/0.677(7).

One of two *p*-xylene molecules is ill defined and partially occupied. The C–CH_3 bonds were restrained to a target value of 1.55(2) Å. The other C–C bonds were restrained to be of similar length, and the entire molecule was restrained to be close to planar. Subject to these conditions the occupancy ratio refined to 0.545(9).

Two of four nitrobenzene molecules were refined as disordered. The other two were refined as partially occupied. All nitrobenzene molecules were restrained to have similar geometries. U_{ij} components of ADPs for disordered atoms closer to each other than 2.0 Å were restrained to be similar, and the atoms of the molecule of O33 were restrained to be close to isotropic. Subject to these conditions the occupancy ratios refined to 0.674(7)/0.326(7) for the molecule O31 and to 0.728(7)/0.272(7) for the molecule of O37. The occupancies refined to 0.673(7) for the molecule of O33, and to 0.580(8) for the molecule of O35.

The structure contains additional 643 Å^3 of solvent accessible voids. No substantial electron density peaks were found in the solvent accessible voids (less than 1.8 electrons per cubic Angstrom) and the residual electron density peaks are not arranged in an interpretable pattern. The structure factors were instead augmented via reverse Fourier transform methods using the SQUEEZE routine as implemented in the program Platon.[1] The resultant FAB file containing the structure factor contribution from the electron content of the void space was used together with the original HKL file in further refinement. The FAB file with details of the SQUEEZE results is appended to the CIF file. The SQUEEZE procedure corrected for 139 electrons within the solvent accessible voids.

2: Refined as a two-component inversion twin. The carbon atoms of one pyrazole ligand (of N15) were refined as disordered. The disordered moieties were restrained to have similar

geometries as another better defined pyrazole ligand, and to be close to planar. U_{ij} components of ADPs for disordered atoms closer to each other than 2.0 Å were restrained to be similar. Subject to these conditions the occupancy ratio refined to 0.719(16)/0.281(16).

Hydroxyl H atom positions were refined and O–H distances were restrained to a target value of 0.84(2) Angstrom. The position of H atom H2O was further restrained based on hydrogen bonding considerations (1.95(2) Å to O10).

The structure is not charge balanced, missing a second cation. Additional void space would be capable to hold a second methyltriphenylphosphonium cation (the solvent accessible volume is 15877 Å^3). This large void at the center of the cell is entirely featureless, with the largest residual electron densities located at the edges and the rim of the void (the largest Q peak, located on a special position at 0.3558/1.0000/0.5000, has a count of 1.28). The structure factors were thus augmented via reverse Fourier transform methods using the SQUEEZE routine as implemented in the program Platon.[1] The resultant FAB file containing the structure factor contribution from the electron content of the void space was used together with the original HKL file in further refinement. The FAB file with details of the SQUEEZE results is appended to the CIF file. The SQUEEZE procedure corrected for 3133 electrons within the solvent accessible voids.

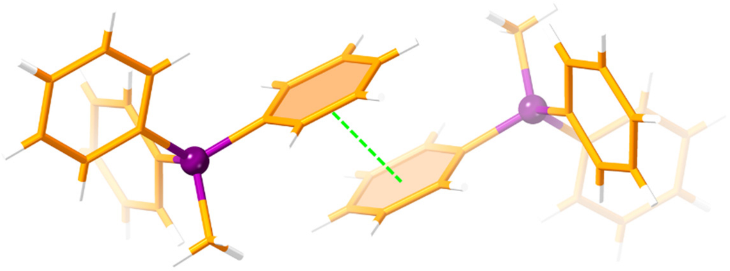

Figure S4. Illustration of the π–π stacking interaction between phenyl rings of the $MePh_3P^+$ counterions within the crystal lattice of **1**.

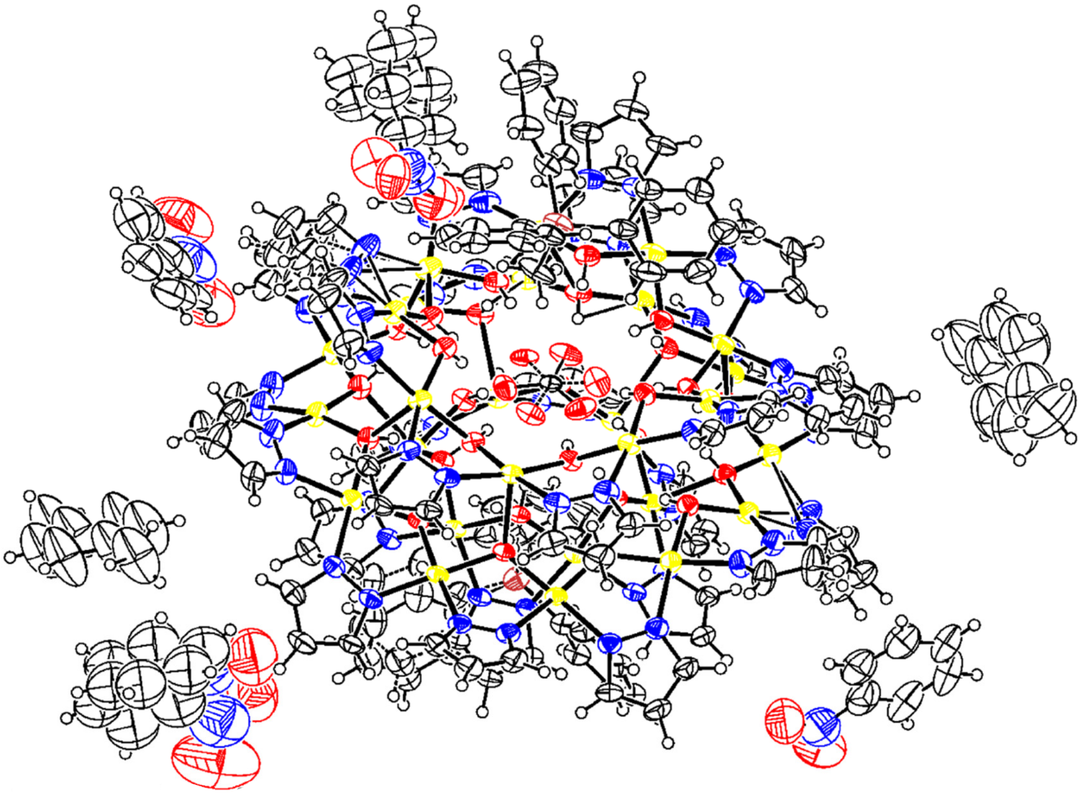

Figure S5. Thermal ellipsoid plot of the crystal structure of **1**.

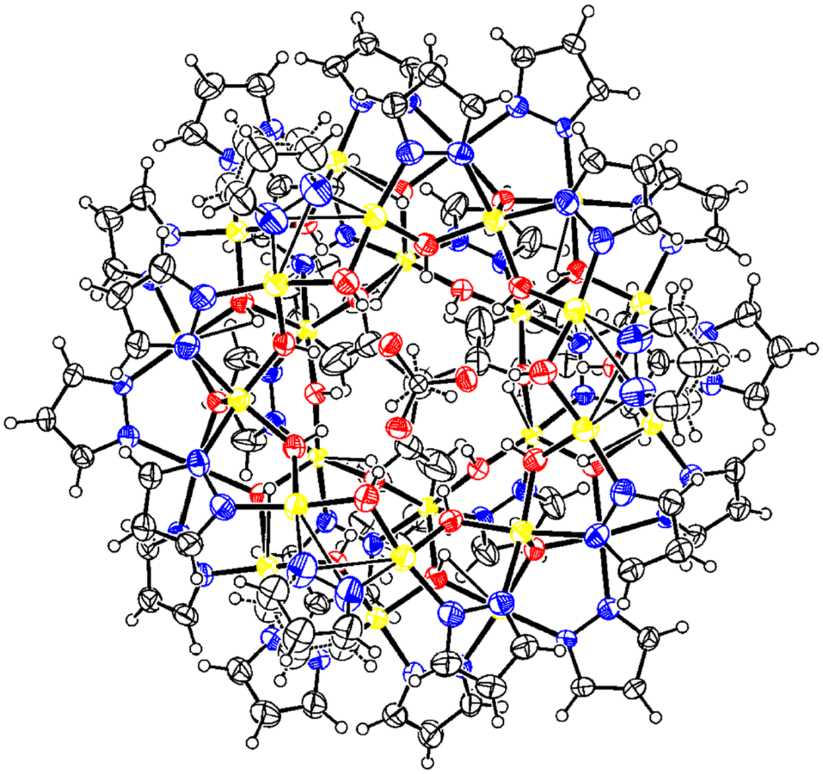

Figure S6. Thermal ellipsoid plot of the crystal structure of **2**.

Table S1. Crystallographic data for the triclinic (**1**) and cubic (**2**) nanojars.

	**1**	**2**
Formula (sum)	$C_{147.88}H_{170.72}Cu_{27}N_{57.25}O_{36.51}P_2$	$C_{101}H_{126}Cu_{27}N_{54}O_{30}P$
Formula (moiety)	$C_{81}H_{108}Cu_{27}N_{54}O_{27}$, CO_3, 2($C_{19}H_{18}P$), 3.253($C_6H_5NO_2$), 1.045(C_8H_{10}), solvent	$C_{81}H_{108}Cu_{27}N_{54}O_{27}$, CO_3, $C_{19}H_{18}P$, cation, solvent
FW (g·mol^{-1})	5112.10	4323.10
Crystal system	Triclinic	Cubic
Space group	$P\bar{1}$ (No. 2)	$I23$ (No. 197)
a (Å)	20.0613(7)	34.526(4)
b (Å)	23.5453(7)	34.526(4)
c (Å)	24.7360(8)	34.526(4)
α (deg)	106.9415(18)	90.000
β (deg)	93.547(2)	90.000
γ (deg)	112.9470(16)	90.000
V (Å^3)	10086.2(6)	41158(14)
Z	2	8
D_{calc} (g·cm^{-3})	1.683	1.395
μ (mm^{-1})	2.871	2.790
θ range (deg)	1.888–28.354	2.503–28.274
Reflns collected	111263	33897
R_{int}	0.0404	0.0295
Obsd reflns [$I > 2\sigma(I)$]	33698	11911
Data/restraints/parameters	49033/2014/2922	14234/194/697
GOF (on F^2)	1.037	1.035
R factors [$I > 2\sigma(I)$]	R_1 = 0.0492<br>wR_2 = 0.1323	R_1 = 0.0352<br>wR_2 = 0.0908
R factors (all data)	R_1 = 0.0784<br>wR_2 = 0.1500	R_1 = 0.0464<br>wR_2 = 0.0972
Maximum peak/hole (e·Å^{-3})	1.211/−0.849	0.408/−0.442
CCDC number	2349706	2349707

Table S2. Comparison of bond lengths (Å) and angles (°), Cu⋯O distances shorter than the sum of the van der Waals radii of Cu and O (2.92 Å) and H-bonding (with D⋯A distances shorter than 3.2 Å) in **1** and **2** (based on the major component in the case of disordered moieties).

	**$Cu_{27}CO_3$ (1)**	**$Cu_{27}CO_3$ (2)**
Cu–O within Cu_n rings	1.907(3)–1.944(3)<br>avg: 1.926(3)	1.907(4)–1.945(4)<br>avg: 1.924(4)
Cu–N within Cu_n rings	1.956(4)–2.010(11)<br>avg: 1.976(4)	1.957(6)–2.018(5)<br>avg: 1.978(5)
N–Cu–O (*trans*) within Cu_{12} ring	165.40(17)–175.7(14)<br>avg: 171.4(2)	167.19(18)–175.93(19)<br>avg: 172.4(2)
N–Cu–O (*trans*) within Cu_9 ring	166.59(16)–176.93(17)<br>avg: 172.4(2)	168.6(2)–175.1(2)<br>avg: 172.4(2)
N–Cu–O (*trans*) within Cu_6 ring	162.97(16)–176.74(14)<br>avg: 169.7(2)	163.93(19)–175.78(17)<br>avg: 169.9(2)
Average of all N–Cu–O (trans) angles	*171.2(2)*	*171.6(2)*
N–Cu–O (*cis*) within Cu_{12} ring	85.50(16)–87.39(14)<br>avg: 86.6(2)	85.48(19)–87.82(18)<br>avg: 86.6(2)
N–Cu–O (*cis*) within Cu_9 ring	83.43(18)–86.38(15)<br>avg: 84.7(2)	83.42(19)–85.8(3)<br>avg: 84.8(2)
N–Cu–O (*cis*) within Cu_6 ring	83.82(14)–85.03(13)<br>avg: 84.5(2)	84.44(17)–84.93(17)<br>avg: 84.8(2)
Average of all N–Cu–O (cis) angles	*85.3(2)*	*85.4(2)*
Cu⋯Cu distances in Cu_{12} ring	3.2030(8)–3.3722(10)<br>avg: 3.302(1)	3.2092(10)–3.3423(10)<br>avg: 3.304(1)
Cu⋯Cu distances in Cu_9 ring	3.1638(10)–3.3624(8)<br>avg: 3.297(1)	3.1525(10)–3.3475(11)<br>avg: 3.277(1)
Cu⋯Cu distances in Cu_6 ring	3.2052(9)–3.3942(11)<br>avg: 3.306(1)	3.2219(10), 3.3768(10)<br>avg: 3.299(1)
Average of all Cu⋯Cu distances in Cu_n rings	*3.302(1)*	*3.293(1)*
Cu⋯O between Cu_9 and Cu_{12} rings	2.396(4)–2.815(4)<br>(5 interactions)<br>avg: 2.562(4)	2.411(4)<br>(3 interactions)<br>avg: 2.411(4)
Cu⋯O between Cu_6 and Cu_{12} rings	2.370(4)–2.477(4)<br>(6 interactions)<br>avg: 2.429(4)	2.412(4), 2.435(4)<br>(6 interactions)<br>avg: 2.424(4)
Average of all Cu⋯O interactions between Cu_n rings	*2.496(4)*<br>*(11 interactions)*	*2.417(4)*<br>*(9 interactions)*
H-bonded O⋯O distances between Cu_9 and Cu_{12} rings	2.743(4)–2.840(5)<br>(6 interactions)<br>avg: 2.781(5)	2.790(6), 2.804(6)<br>(6 interactions)<br>avg: 2.797(6)
H-bonded O⋯O distances between Cu_6 and Cu_{12} rings	2.722(5)–2.755(5)<br>(6 interactions)<br>avg: 2.739(5)	2.722(5), 2.748(5)<br>(6 interactions)<br>avg: 2.735(5)
Average of all H-bonded O⋯O distances between Cu_n rings	*2.760(5)*<br>*(12 interactions)*	*2.766(6)*<br>*(12 interactions)*
H-bonded O⋯O distances between Cu_n rings and CO_3^{2-}	2.724(5)–3.120(6)<br>(13 interactions)<br>avg: 2.880(6)	2.785(6)–2.869(6)<br>(12 interactions)<br>avg: 2.838(6)

Table S3. Comparison of the dihedral, twist and fold angles (°) between pyrazolate moieties and adjacent Cu–O–Cu units in **1** and **2** (based on the major component in the case of disordered moieties).

	DIHEDRAL ANGLE	TWIST ANGLE	FOLD ANGLE
**$Cu_{27}CO_3$ (1)** Cu_{12}-ring	31.4(9)–57.3(3)<br>avg: 43.5(3)	0.1(2)–9.7(8)<br>avg: 4.2(2)	27.5(16)–57.2(3)<br>avg: 42.6(3)
**$Cu_{27}CO_3$ (1)** Cu_9-ring	32.5(8)–60.1(3)<br>avg: 52.3(3)	1.0(2)–6.3(7)<br>avg: 3.0(2)	32.0(8)–60.1(3)<br>avg: 52.3(3)
**$Cu_{27}CO_3$ (1)** Cu_6-ring	26.8(3)–67.8(2)<br>avg: 49.4(3)	0.91(16)–10.30(17)<br>avg: 4.0(2)	26.0(3)–67.8(2)<br>avg: 49.8(3)
Avg. **$Cu_{27}CO_3$ (1)**	*48.4(3)*	*3.7(2)*	*48.2(3)*
**$Cu_{27}CO_3$ (2)** Cu_{12}-ring	35.8(3)–56.5(3)<br>avg: 41.8(3)	1.2(2)–11.34(19)<br>avg: 6.7(2)	34.6(3)–56.4(3)<br>avg: 42.6(3)
**$Cu_{27}CO_3$ (2)** Cu_9-ring	46.8(3)–64.8(7)<br>avg: 55.6(5)	0.1(2)–6.6(3)<br>avg: 2.3(5)	38.2(5)–64.8(7)<br>avg: 52.8(5)
**$Cu_{27}CO_3$ (2)** Cu_6-ring	29.1(4), 70.3(3)<br>avg: 49.7(4)	0.2(3), 1.7(2)<br>avg: 1.0(3)	29.1(4), 70.2(3)<br>avg: 49.7(4)
Avg. **$Cu_{27}CO_3$ (2)**	*49.0(4)*	*3.3(3)*	*49.9(4)*

Table S4. Comparison of the dihedral, twist and fold angles (°) between adjacent pyrazolate moieties in **1** and **2** (based on the major component in the case of disordered moieties).

	DIHEDRAL ANGLE	TWIST ANGLE	FOLD ANGLE	CENTROID-CENTROID DISTANCE	
				**D. A. > 35°**	**D. A. < 35°**
**$Cu_{27}CO_3$ (1)** Cu_{12}-ring	41.3(9)–70.3(9)<br>avg: 57.3(3)	40.9(10)–69.1(9)<br>avg: 57.6(3)	3.1(3)–37.7(5)<br>avg: 19.7(3)	4.764(5)–4.929(11)<br>avg. of 12: 4.861(5)	–
**$Cu_{27}CO_3$ (1)** Cu_9-ring	13.8(2)–49.5(2)<br>avg: 34.6(3)	0.6(3)–48.0(2)<br>avg: 31.6(3)	1.7(8)–37.4(6)<br>avg: 17.9(6)	4.927(4)–5.011(4)<br>avg of 6: 4.969(4)	5.103(8)–5.175(3)<br>avg of 3: 5.149(4)
**$Cu_{27}CO_3$ (1)** Cu_6-ring	37.6(3)–56.0(2)<br>avg: 45.7(3)	25.7(2)–37.4(3)<br>avg: 31.9(3)	19.9(2)–64.6(11)<br>avg: 41.9(3)	4.909(4)–5.034(4)<br>avg of 6: 4.964(4)	–
Avg. **$Cu_{27}CO_3$ (1)**	*45.9(3)*	*40.4(3)*	*26.5(3)*	*4.931(4)*	*5.149(4)*
**$Cu_{27}CO_3$ (2)** Cu_{12}-ring	42.2(3)–63.5(3)<br>avg: 53.4(3)	43.7(3)–61.4(3)<br>avg: 52.4(3)	4.9(3)–46.7(5)<br>avg: 23.5(3)	4.830(4)–4.871(4)<br>avg. of 12: 4.845(4)	–
**$Cu_{27}CO_3$ (2)** Cu_9-ring	17.3(3)–49.0(7)<br>avg: 36.1(7)	11.2(3)–49.8(7)<br>avg: 34.3(7)	5.1(8)–32.1(16)<br>avg: 16.8(8)	4.950(6), 5.015(6)<br>avg of 6: 4.983(6)	5.146(4)<br>avg of 3: 5.146(4)
**$Cu_{27}CO_3$ (2)** Cu_6-ring	47.7(4), 51.1(4)<br>avg: 49.4(4)	38.3(4), 40.2(4)<br>avg: 39.3(4)	30.8(4), 63.0(9)<br>avg: 46.9(4)	4.977(4), 4.986(4)<br>avg of 6: 4.982(4)	–
Avg. **$Cu_{27}CO_3$ (2)**	*46.3(4)*	*42.0(4)*	*29.1(4)*	*4.937(4)*	*5.146(4)*

Table S5. Comparison of the deviations (Å) of Cu atoms in different Cu_x rings from the Cu_x mean-planes in **1** and **2**.

	**Deviation from Cu_m mean-plane**
**$Cu_{27}CO_3$ (1)** Cu_{12}-ring	0.061–0.473<br>avg: 0.275
**$Cu_{27}CO_3$ (1)** Cu_9-ring	0.052–0.574<br>avg: 0.355
**$Cu_{27}CO_3$ (1)** Cu_6-ring	0.001–0.072<br>avg: 0.036
Avg. **$Cu_{27}CO_3$ (1)**	*0.222*
**$Cu_{27}CO_3$ (2)** Cu_{12}-ring	0.301–0.390<br>avg: 0.346
**$Cu_{27}CO_3$ (2)** Cu_9-ring	0.269–0.572<br>avg: 0.381
**$Cu_{27}CO_3$ (2)** Cu_6-ring	0.002, 0.002<br>avg: 0.002
Avg. **$Cu_{27}CO_3$ (2)**	*0.243*

Table S6. Selected bond lengths (Å) for **1** (Cu1–Cu6: Cu_6-ring; Cu7–Cu18: Cu_{12}-ring; Cu19–Cu27: Cu_9-ring; O28–O30, O28B–O30B: CO_3^{2-} anion disordered over two positions in an 81/18 ratio).

C82–O28 1.287(5)	Cu6–N10 1.993(4)	Cu14–O14 1.916(3)	Cu21–O21 1.927(3)
C82–O29 1.277(6)	Cu6–N11 1.991(4)	Cu14–N26 1.958(4)	Cu21–N40 2.010(4)
C82–O30 1.257(6)	Cu7–O7 1.933(3)	Cu14–N27 1.963(4)	Cu21–N41 2.021(11)
C82B–O28B 1.277(13)	Cu7–O18 1.917(3)	Cu15–O14 1.915(3)	Cu21–N41B 1.977(9)
C82B–O29B 1.282(13)	Cu7–N13 1.965(4)	Cu15–O15 1.927(3)	Cu22–O21 1.907(3)
C82B–O30B 1.268(14)	Cu7–N36 1.974(4)	Cu15–N28 1.972(4)	Cu22–O22 1.909(3)
Cu1–O1 1.925(3)	Cu8–O7 1.936(3)	Cu15–N29 1.959(4)	Cu22–N42 1.957(11)
Cu1–O6 1.937(3)	Cu8–O8 1.927(3)	Cu16–O15 1.930(3)	Cu22–N42B 1.968(8)
Cu1–N1 1.983(4)	Cu8–N14 1.980(4)	Cu16–O16 1.919(3)	Cu22–N43 1.959(4)
Cu1–N12 1.993(4)	Cu8–N15 1.956(4)	Cu16–N30 1.974(4)	Cu23–O22 1.921(3)
Cu2–O1 1.937(3)	Cu9–O8 1.931(3)	Cu16–N31 1.967(4)	Cu23–O23 1.933(3)
Cu2–O2 1.939(3)	Cu9–O9 1.923(3)	Cu17–O16 1.924(3)	Cu23–N44 1.970(4)
Cu2–O9 2.370(3)	Cu9–N16 1.962(4)	Cu17–O17 1.929(3)	Cu23–N45 1.983(4)
Cu2–N2 1.994(4)	Cu9–N17 1.979(5)	Cu17–N32 1.972(4)	Cu24–O23 1.938(3)
Cu2–N3 2.001(4)	Cu10–O9 1.921(4)	Cu17–N33 1.98(2)	Cu24–O24 1.937(3)
Cu3–O2 1.934(3)	Cu10–O10 1.919(3)	Cu17–N33B 1.96(2)	Cu24–N46 2.009(4)
Cu3–O3 1.925(3)	Cu10–N18 1.958(4)	Cu18–O17 1.926(3)	Cu24–N47 2.004(4)
Cu3–N4 1.981(4)	Cu10–N19 1.963(5)	Cu18–O18 1.916(3)	Cu24–O14 2.428(3)
Cu3–N5 1.990(4)	Cu11–O10 1.912(3)	Cu18–N34 1.968(18)	Cu25–O24 1.915(3)
Cu4–O3 1.937(3)	Cu11–O11 1.933(3)	Cu18–N34B 1.936(18)	Cu25–O25 1.914(3)
Cu4–O4 1.944(3)	Cu11–N20 1.970(4)	Cu18–N35 1.990(4)	Cu25–N48 1.962(4)
Cu4–O13 2.395(3)	Cu11–N21 1.964(4)	Cu19–O19 1.907(3)	Cu25–N49 1.957(4)
Cu4–N6 1.998(4)	Cu12–O11 1.930(3)	Cu19–O27 1.909(3)	Cu26–O25 1.911(3)
Cu4–N7 1.988(4)	Cu12–O12 1.921(3)	Cu19–N37 1.968(4)	Cu26–O26 1.922(3)
Cu5–O4 1.923(3)	Cu12–N22 1.976(4)	Cu19–N54 1.956(4)	Cu26–N50 1.969(4)
Cu5–O5 1.938(3)	Cu12–N23 1.962(4)	Cu20–O19 1.922(3)	Cu26–N51 1.988(4)
Cu5–O15 2.434(3)	Cu13–O12 1.938(3)	Cu20–O20 1.921(3)	Cu27–O18 2.396(3)
Cu5–N8 1.985(4)	Cu13–O13 1.917(3)	Cu20–N38 1.980(4)	Cu27–O26 1.938(3)
Cu5–N9 1.983(4)	Cu13–N24 1.958(4)	Cu20–N39 1.973(5)	Cu27–O27 1.927(3)
Cu6–O5 1.926(3)	Cu13–N25 1.980(4)	Cu21–O10 2.404(4)	Cu27–N52 2.008(4)
Cu6–O6 1.941(3)	Cu14–O13 1.917(3)	Cu21–O20 1.936(3)	Cu27–N53 1.993(4)
Cu6–O17 2.421(3)			

Table S7. Hydrogen bonding data for **1** (O1–O6: Cu_6-ring; O7–O18: Cu_{12}-ring; O19–O27: Cu_9-ring; O28–O30 and O28B–O30B: CO_3^{2-} anion disordered over two positions in an 81/18 ratio).

D*—H···*A	***D*—H (Å)**	**H···*A* (Å)**	***D*···*A* (Å)**	***D*—H···*A* (°)**
O1—H1O···O28	0.83(2)	1.95(3)	2.751(5)	162(5)
O1—H1O···O28B	0.83(2)	2.24(4)	3.007(19)	154(5)
O2—H2O···O29	0.83(2)	2.35(3)	3.120(6)	155(5)
O2—H2O···O28B	0.83(2)	2.00(3)	2.792(17)	161(6)
O3—H3O···O29	0.825(19)	1.93(3)	2.724(5)	161(5)
O3—H3O···O29B	0.825(19)	2.27(3)	3.05(2)	157(5)
O4—H4O···O30	0.816(19)	2.59(3)	3.353(6)	156(5)
O4—H4O···O29B	0.816(19)	1.98(3)	2.789(17)	171(5)
O5—H5O···O30	0.827(19)	1.96(3)	2.752(5)	161(5)
O5—H5O···O29B	0.827(19)	2.62(3)	3.42(2)	163(5)
O5—H5O···O30B	0.827(19)	2.50(4)	3.22(2)	146(5)
O6—H6O···O28	0.83(2)	2.51(3)	3.242(6)	148(5)
O6—H6O···O30	0.83(2)	2.62(3)	3.410(6)	161(5)
O6—H6O···O30B	0.83(2)	2.11(3)	2.92(2)	165(5)
O7—H7O···O27	0.84(2)	2.02(3)	2.816(5)	158(6)
O8—H8O···O1	0.812(17)	1.973(16)	2.755(4)	161(5)
O9—H9O···O20	0.84(2)	1.91(2)	2.743(4)	173(6)
O10—H10O···O2	0.82(2)	1.96(3)	2.724(4)	154(6)
O11—H11O···O21	0.84(2)	2.01(2)	2.840(5)	175(6)
O12—H12O···O3	0.822(16)	1.932(16)	2.722(4)	161(5)
O13—H13O···O23	0.826(19)	1.93(2)	2.747(4)	168(5)
O14—H14O···O4	0.84(2)	1.90(2)	2.742(4)	178(5)
O15—H15O···O24	0.820(19)	1.97(2)	2.783(4)	171(6)
O16—H16O···O5	0.81(2)	1.93(2)	2.737(4)	169(6)
O17—H17O···O26	0.827(19)	1.99(3)	2.756(4)	155(6)
O18—H18O···O6	0.824(19)	1.95(3)	2.754(4)	164(6)
O19—H19O···O28	0.83(2)	1.97(2)	2.789(5)	170(6)
O19—H19O···O28B	0.83(2)	2.57(4)	3.314(19)	150(5)
O20—H20O···O28	0.82(2)	2.23(2)	3.028(6)	165(6)
O20—H20O···O28B	0.82(2)	1.82(3)	2.612(13)	163(6)
O21—H21O···O29	0.83(2)	2.04(2)	2.862(6)	172(6)
O21—H21O···O28B	0.83(2)	2.11(4)	2.845(18)	148(6)
O22—H22O···O29	0.829(19)	1.96(2)	2.784(5)	174(6)
O22—H22O···O29B	0.829(19)	2.49(4)	3.25(2)	154(5)
O23—H23O···O29	0.811(19)	2.30(2)	3.087(6)	165(5)
O23—H23O···O29B	0.811(19)	1.83(3)	2.620(15)	164(5)
O24—H24O···O30	0.862(16)	2.038(16)	2.877(6)	164(5)
O24—H24O···O29B	0.862(16)	2.07(3)	2.881(19)	156(4)
O25—H25O···O30	0.83(2)	2.00(2)	2.807(5)	166(6)
O25—H25O···O30B	0.83(2)	2.37(3)	3.16(2)	160(5)
O26—H26O···O30	0.83(2)	2.24(3)	3.012(6)	156(6)
O26—H26O···O30B	0.83(2)	1.72(3)	2.540(15)	175(6)
O27—H27O···O28	0.841(18)	2.02(2)	2.849(6)	170(5)
O27—H27O···O30B	0.841(18)	2.109(19)	2.854(18)	148 (4)

Table S8. Selected bond lengths (Å) for **2** (Cu1, Cu2: Cu_6-ring; Cu3–Cu6: Cu_{12}-ring; Cu7–Cu9: Cu_9-ring; O10: CO_3^{2-} anion). Symmetry operations: *i)* −y+1, z, −x+1; *ii)* −z+1, −x+1, y.

C28–O10 1.279(4)	Cu3–O3 1.918(4)	Cu5–N9 1.980(5)	Cu8–O7 1.907(4)
Cu1–O1 1.934(4)	Cu3–O6 1.918(4)	Cu6–O5 1.921(4)	Cu8–O8 1.919(5)
Cu1–O2^{i} 1.928(4)	Cu3–N5 1.970(4)	Cu6–O6^{ii} 1.918(4)	Cu8–N14 1.960(6)
Cu1–N1 1.984(5)	Cu3–N12^{i} 1.966(5)	Cu6–N10 1.967(5)	Cu8–N15 1.957(6)
Cu1–N4^{i} 1.977(4)	Cu4–O3 1.939(4)	Cu6–N11 1.965(4)	Cu8–N15B 1.957(6)
Cu1–O5^{i} 2.435(4)	Cu4–O4 1.925(4)	Cu7–O7 1.945(4)	Cu9–O8 1.909(5)
Cu2–O1 1.934(4)	Cu4–N6 1.979(5)	Cu7–O9^{i} 1.931(4)	Cu9–O9 1.917(4)
Cu2–O2 1.926(4)	Cu4–N7 1.990(5)	Cu7–N13 2.011(5)	Cu9–N16 1.979(6)
Cu2–N2 1.987(5)	Cu5–O4 1.917(4)	Cu7–N18^{i} 2.018(5)	Cu9–N16B 1.979(6)
Cu2–N3 1.973(4)	Cu5–O5 1.928(4)	Cu7–O6 2.412(4)	Cu9–N17 1.974(6)
Cu2–O3 2.412(4)	Cu5–N8 1.972(5)		

Table S9. Hydrogen bonding data for **2** (O1, O2: Cu_6-ring; O3–O6: Cu_{12}-ring; O7–O9: Cu_9-ring; O10: CO_3^{2-} anion).

D*—H···*A	***D*—H (Å)**	**H···*A* (Å)**	***D*···*A* (Å)**	***D*—H···*A* (°)**
O1—H1O···O10	0.82(3)	2.65(4)	3.431(6)	159(6)
O2—H2O···O10	0.87(2)	1.99(2)	2.785(6)	152(5)
O3—H3O···O7	0.82(3)	2.03(4)	2.804(6)	159(7)
O4—H4O···O2	0.85(2)	1.92(3)	2.748(5)	166(7)
O5—H5O···O9	0.83(3)	2.02(4)	2.790(6)	155(7)
O6—H6O···O1	0.86(3)	1.87(3)	2.723(5)	172(8)
O7—H7O···O10	0.85(3)	2.09(4)	2.869(6)	152(7)
O8—H8O···O10	0.84(3)	2.06(5)	2.833(6)	154(8)
O9—H9O···O10	0.83(3)	2.07(4)	2.866(6)	160(8)

3. NMR SPECTROSCOPIC DATA

Table S10. Variable-temperature ^{1}H NMR chemical shifts (ppm) in DMSO-d_6 of **Cu_nCO_3** nanojars. Missing values are due to lack of significant amounts of a particular species in the mixture and/or to inability of unambiguous assignment due to overlap or excessive broadening.

NANOJAR	22 °C	30 °C	40 °C	50 °C	60 °C	70 °C	80 °C	90 °C	100 °C	110 °C	120 °C	130 °C	140 °C	150 °C
**Cu$_{27}$ (6+12+9)**														
Cu$_9$ ring, pz-4-*H*	37.63	37.23	36.75	36.29	35.84	35.41	35.00	34.59	34.20	33.82	33.44	33.09	32.72	32.39
Cu$_9$ ring, pz-3,5-*H*$_2$	33.24	32.88	32.45	32.04	31.64	31.26	30.89	30.53	30.17	29.83	29.50	29.19	28.85	28.56
Cu$_6$ ring, pz-4-*H*	31.26	31.34	31.41	31.44	31.45	31.43	31.39	31.34	31.27	31.18	31.09	31.00	30.88	30.76
Cu$_6$ ring, pz-3,5-*H*$_2$	28.24	28.30	28.36	28.40	28.43	28.47	~28.4	~28.3	~28.2	28.16	28.09	28.00	27.89	27.78
Cu$_{12}$ ring, pz-4-*H(9)*	27.77	27.81	27.84	27.87	27.89	27.90	27.90	27.89	27.87	27.85	27.82	27.78	27.74	27.69
Cu$_{12}$ ring, pz-4-*H(6)*	26.35	26.39	26.42	26.44	26.46	26.47	26.47	26.46	26.43	26.40	26.37	26.33	26.28	26.23
Cu$_{12}$ ring, pz-3,5-*H*$_2$*(9)*	22.45	22.47	22.49	22.49	22.50	22.49	22.48	22.46	22.44	22.41	22.37	22.34	22.29	22.25
Cu$_{12}$ ring, pz-3,5-*H*$_2$*(6)*	22.43	22.45	22.46	22.6	22.47	22.46	22.45	22.43	22.40	22.37	22.33	22.30	22.25	22.20
Cu$_{12}$ ring, O*H(6)*	−29.65	−29.69	−29.72	−29.73	−29.72	−29.69	−29.64	−29.66	−29.50	−29.40	−29.30	−29.14	~−29.0	~−28.9
Cu$_{12}$ ring, O*H(9)*	−32.50	−32.77	−33.05	−33.25	−33.41	−33.53	−33.63	−33.76	−33.69	−33.71	−33.70	−33.66	−33.61	−33.55
Cu$_6$ ring, O*H*	− 41.25	−41.30	−41.34	−41.35	−41.35	−41.33	−41.28	−41.21	−41.11	−41.00	−40.86	−40.72	−40.59	−40.35
Cu$_9$ ring, O*H*	−68.08	−66.96	−65.63	−64.38	−63.19	−62.04	−60.95	−59.91	−58.90	−57.90	−56.98	−56.07	−55.21	−54.36
**Cu$_{29}$ (7+13+9)**														
Cu$_9$ ring, pz-4-*H*	33.87	33.68	33.44	33.19	32.95	32.70	32.46	32.23	31.99	31.76	31.51	31.29	31.05	30.81
Cu$_9$ ring, pz-3,5-*H*$_2$	29.79	29.62	29.42	29.21	29.00	28.79	28.59	~28.3	~28.2	27.96	~27.8	27.57	27.36	27.16
Cu$_7$ ring, pz-4-*H*	30.81	30.84	30.85	30.85	30.84	30.81	30.76	30.71	30.65	30.58	30.49	30.42	30.32	30.22
Cu$_7$ ring, pz-3,5-*H*$_2$	27.41	27.43	27.44	27.43	27.42	27.38	27.35	27.30	27.24	27.18	27.10	27.02	26.93	26.88
Cu$_{13}$ ring, pz-4-*H*	27.12	27.18	27.24	27.30	27.34	27.38	27.40	27.42	27.43	27.42	27.41	27.40	27.37	27.33
Cu$_{13}$ ring, pz-3,5-*H*$_2$	22.02	22.05	22.08	22.11	22.13	22.14	22.15	22.15	22.14	22.13	22.11	22.09	22.06	22.02
Cu$_{13}$ ring, O*H*	−29.32	−29.37	−29.42	−29.47	−29.53	−29.56	~−29.6	~−29.6	~−29.5	~−29.4	~−29.3	–	–	–
Cu$_7$ ring, O*H*	−36.88	−36.83	−36.83	−36.65	−36.53	−36.42	−36.27	−36.13	−35.96	−35.81	−35.64	–	–	–
Cu$_9$ ring, O*H*	−47.36	−46.88	−46.28	−45.68	−45.09	−44.51	−43.95	−43.40	−42.85	−42.31	−41.79	–	–	–
**Cu$_{29}$ (8+13+8)**														
Cu$_8$ ring, pz-4-*H*	28.52	28.51	28.49	28.47	28.44	28.41	28.37	28.33	28.28	28.23	28.18	28.13	28.06	27.99
Cu$_8$ ring, pz-3,5-*H*$_2$	25.58	25.58	25.56	25.54	25.52	25.49	25.45	25.41	25.37	25.32	25.27	25.22	25.15	25.08
Cu$_{13}$ ring, pz-4-*H*	25.91	26.00	26.11	26.20	26.28	26.36	26.42	26.47	26.51	26.54	26.55	26.56	26.56	26.56
Cu$_{13}$ ring, pz-3,5-*H*$_2$	21.21	21.27	21.33	21.39	21.43	21.47	21.50	21.53	21.54	21.55	21.54	21.54	21.52	21.50
Cu$_{13}$ ring, O*H*	−26.70	−26.91	−27.14	−27.34	−27.53	−27.74	−27.92	−28.10	−28.25	−28.39	−28.54	−28.65	−28.78	−28.86
Cu$_8$ ring, O*H*	−33.61	−33.34	−33.05	−32.81	−32.53	−32.29	−32.05	−31.83	−31.63	−31.44	−31.27	−31.10	−30.96	−30.81
**Cu$_{31}$ (8+14+9)**														
Cu$_9$ ring, pz-4-*H*	–	–	33.86	33.64	33.43	33.21	33.00	32.78	32.56	32.34	32.13	31.92	–	–
Cu$_9$ ring, pz-3,5-*H*$_2$	–	–	~29.3	~29.1	~29.0	~28.8	~28.6	~28.3	~28.1	~28.0	~27.8	27.57	–	–
Cu$_8$ ring, pz-4-*H*	–	–	~30.9	~30.85	30.75	30.66	30.58	~30.5	~30.4	30.30	30.20	30.11	–	–
Cu$_8$ ring, pz-3,5-*H*$_2$	–	–	~27.1	27.09	27.02	26.96	26.89	26.82	26.74	~26.6	~26.5	~26.5	–	–
Cu$_{14}$ ring, pz-4-*H*	28.24	28.30	28.36	28.41	28.44	28.46	28.48	28.48	28.47	28.45	28.43	28.40	–	–
Cu$_{14}$ ring, pz-3,5-*H*$_2$	22.66	22.68	22.70	22.71	22.71	22.70	22.69	22.67	22.65	22.61	22.57	22.53	–	–
Cu$_{14}$ ring, O*H*	–	–	–	–	–	–	–	–	–	–	–	–	–	–
Cu$_8$ ring, O*H*	–	–	–	–	–	–	–	–	–	–	–	–	–	–
Cu$_9$ ring, O*H*	–	–	–	–	–	–	–	–	–	–	–	–	–	–

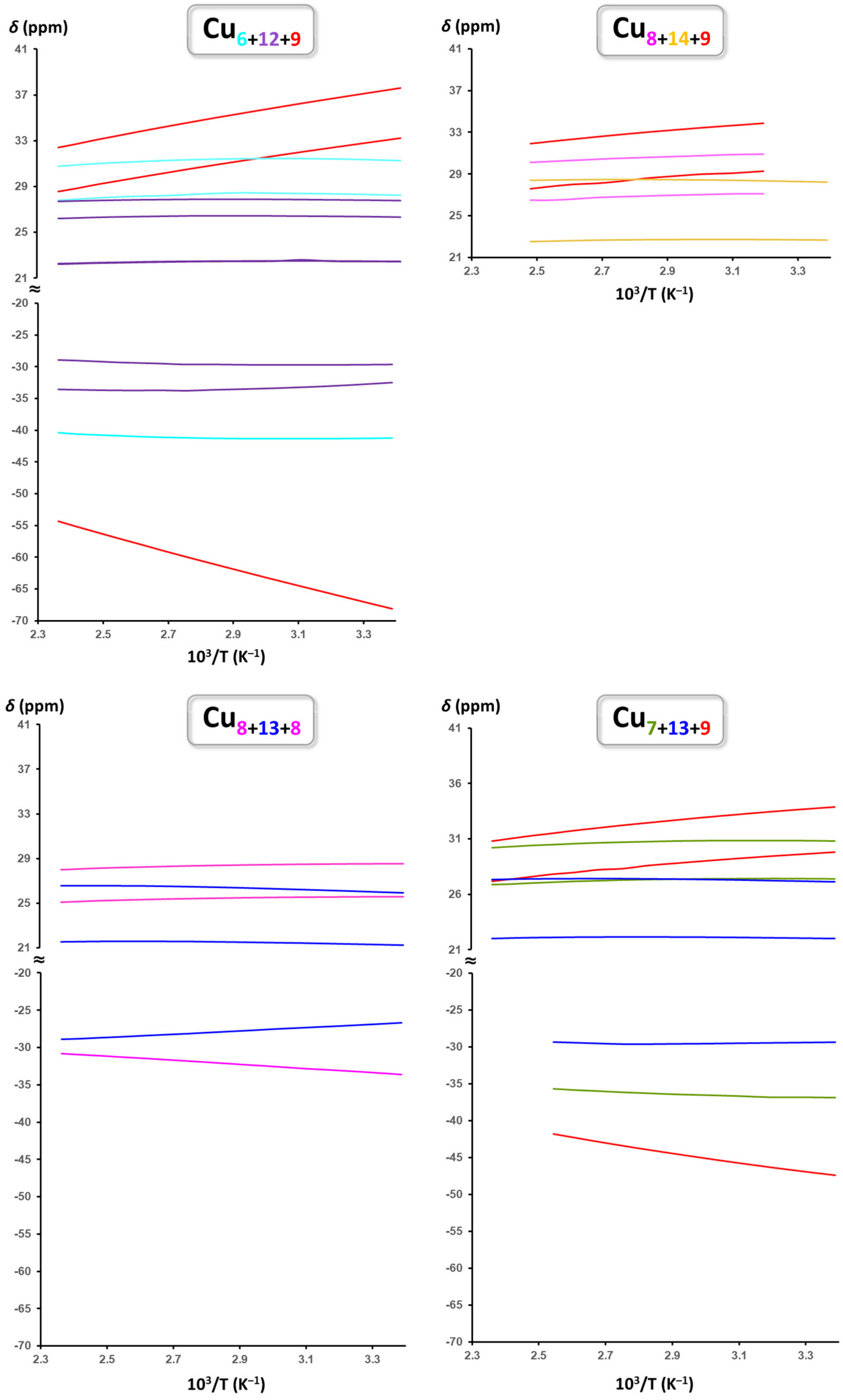

Figure S7. Curie plots for different Cu_x ring protons in the various Cu_nCO_3 nanojars in DMSO-d_6, illustrating the influence of the size of the ring on chemical shift, as well as the difference in chemical shift for the same Cu_x-ring in different nanojars.

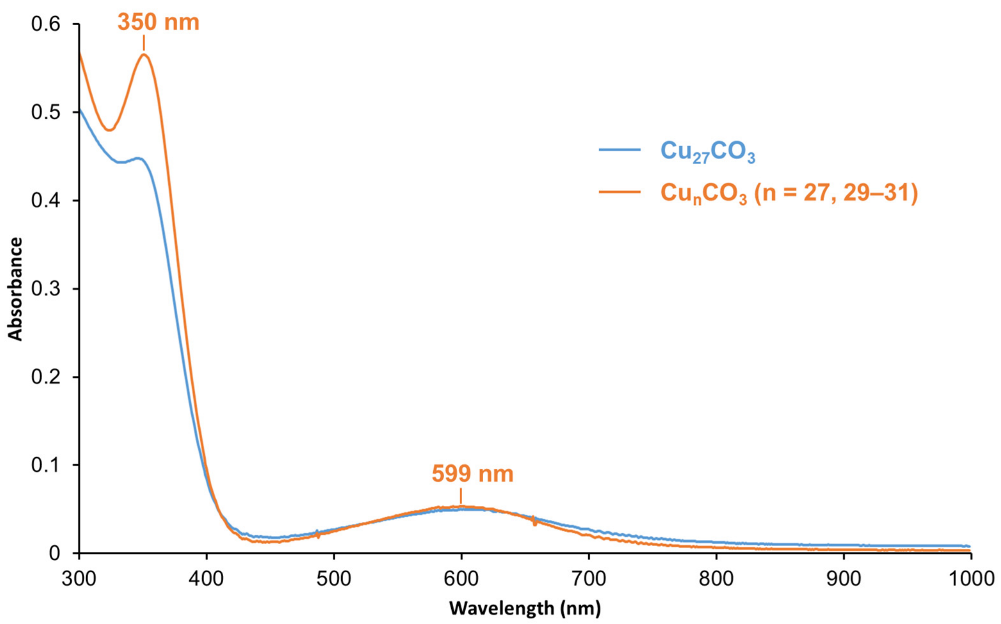

Figure S8. UV-vis spectra of $Cu_{27}CO_3$ and Cu_nCO_3 (n = 27, 29–31) in THF (20 μM).

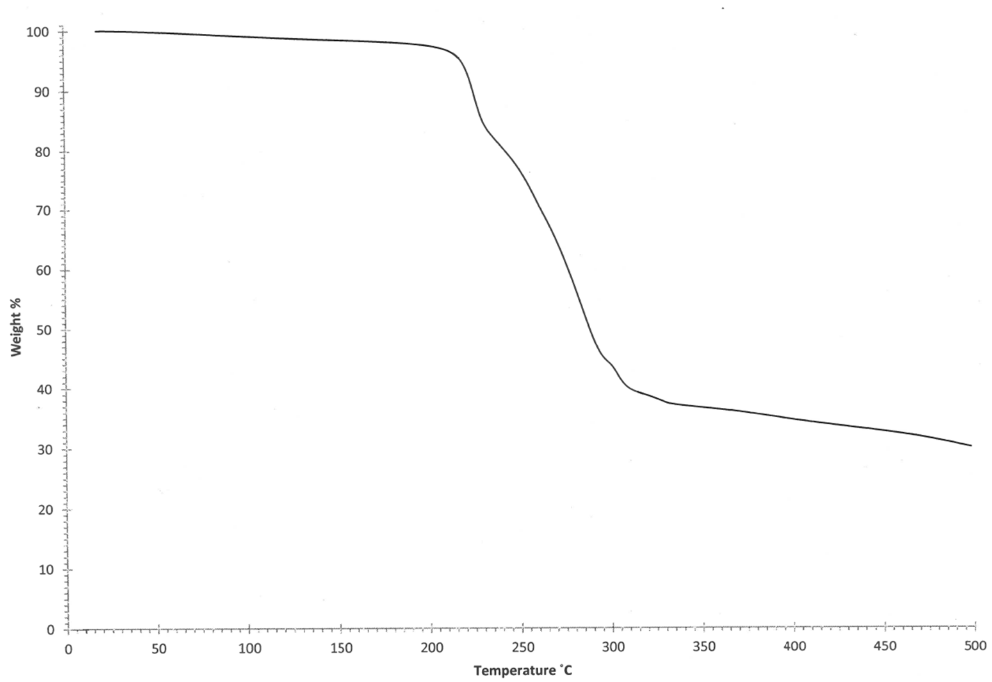

Figure S9. Thermogravimetric analysis (TGA) plot for $Cu_{27}CO_3$ (heated at 5 °C/min under N_2).

4. REFERENCES

[1] Spek, A. L., PLATON SQUEEZE: a Tool for the Calculation of the Disordered Solvent Contribution to the Calculated Structure Factors. *Acta Cryst.* **2015**, *C71*, 9–18.
